# Hydrogels for Tissue Engineering: Addressing Key Design Needs Toward Clinical Translation

**DOI:** 10.3389/fbioe.2022.849831

**Published:** 2022-05-05

**Authors:** Fei Xu, Chloe Dawson, Makenzie Lamb, Eva Mueller, Evan Stefanek, Mohsen Akbari, Todd Hoare

**Affiliations:** ^1^ Department of Chemical Engineering, McMaster University, Hamilton, ON, Canada; ^2^ Department of Mechanical Engineering, University of Victoria, Victoria, BC, Canada; ^3^ Center for Advanced Materials and Related Technologies, University of Victoria, Victoria, BC, Canada; ^4^ Biotechnology Center, Silesian University of Technology, Gliwice, Poland

**Keywords:** Hydrogels, Tissue Engineering, Bioprinting, Electrospinning, Biomaterials

## Abstract

While the soft mechanics and tunable cell interactions facilitated by hydrogels have attracted significant interest in the development of functional hydrogel-based tissue engineering scaffolds, translating the many positive results observed in the lab into the clinic remains a slow process. In this review, we address the key design criteria in terms of the materials, crosslinkers, and fabrication techniques useful for fabricating translationally-relevant tissue engineering hydrogels, with particular attention to three emerging fabrication techniques that enable simultaneous scaffold fabrication and cell loading: 3D printing, *in situ* tissue engineering, and cell electrospinning. In particular, we emphasize strategies for manufacturing tissue engineering hydrogels in which both macroporous scaffold fabrication and cell loading can be conducted in a single manufacturing step – electrospinning, 3D printing, and *in situ* tissue engineering. We suggest that combining such integrated fabrication approaches with the lessons learned from previously successful translational experiences with other hydrogels represents a promising strategy to accelerate the implementation of hydrogels for tissue engineering in the clinic.

## Graphical Abstract

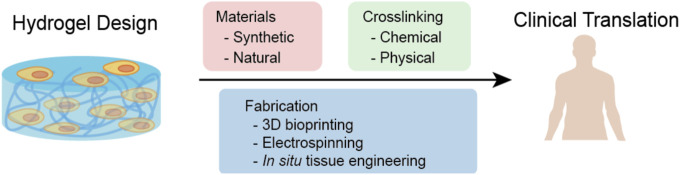

## 1 Introduction

Every year, a high number of deaths or disabilities results from the loss or damage of tissues and organs from injuries or diseases (Chapekar, 2000; Vos et al., 2020). According to the recent international report from the Global Observatory on Donation and Transplantation (GODT), >153,000 organs were transplanted worldwide in 2019, a 4.8% of increase over 2018; however, this number still represented only 10% of global needs. (Organ Donation and Transplantation Activities, 2021) While the transplantation of the damaged tissues or organs either with compatible donors or artificial devices can in part address this challenge (Lee and Mooney, 2001), the number of suitable donors is extremely limited, resulting in long-term wait lists for the patients. Side effects caused by immune/inflammatory responses to transplanted tissues pose additional challenges (Wang et al., 2007; Beyar, 2011; Black et al., 2018). Tissue engineering approaches, first defined in 1988 as the “application of the principles and methods of engineering and life sciences toward a fundamental understanding of structure-function relationship in normal and pathological mammalian tissues and the development of biological substitutes for the repair or regeneration of tissue or organ function”. (Skalak and Fox, 1988; Langer and Vacanti, 1993), aim to address these challenges by fabricating biomaterials into structural scaffolds to mimic the extracellular matrix of cells and provide support for cell proliferation and tissue regeneration; subsequent implantation of these scaffolds, either cell-free (to promote cell ingrowth from the native tissue) or cell-laden (to functionally regenerate native tissue with transplanted cells) leads to tissue regeneration, with scaffolds in most cases designed to degrade at a rate suitable to support new functional tissue formation for as long as required to enable native cell proliferation/organization but ultimately clear once the natively-produced ECM can support the tissue. More recently, tissue engineering has been more specifically defined as the use of cells, scaffolds, and growth factors to replace or regenerate damaged tissues, differentiating it from the broader field of regenerative medicine in which other strategies including gene therapy, cell-based therapies, and/or immunomodulation are leveraged in combination with tissue engineering strategies to regenerate tissues and/or organs (Frey et al., 2016; Han et al., 2020).

Hydrogels, networks of water-soluble polymers physically or chemically crosslinked to form a gel, have been widely used as vehicles to deliver cells to a designated location in the body (Hunt et al., 2014), scaffolds to encapsulate cells to improve cell adhesion or cell proliferation (Ayala et al., 2011), or fillers to fill defects and promote healing while preventing infection (Mooney and Vandenburgh, 2008). The use of hydrogels in this context is motivated by their soft biomechanics mimicking those of native soft tissues such as skin, muscle, fat, or nerve (Ma et al., 2003; Bian et al., 2009; Tibbitt and Anseth, 2009; Guillame-Gentil et al., 2010), tunable pore sizes, compatibility with the cellular environment, suppression of inflammatory responses, and ease of functionalization (Lee and Mooney, 2001; Drury and Mooney, 2003). However, challenges still exist that limit the use of hydrogels as functional tissue engineering scaffolds: 1) the mismatch between the pore (mesh) size of the gel network of conventional hydrogels (on the tens of nanometer scale) and the dimensions of cells (on the micron scale) poses challenges with promoting cell proliferation unless specific strategies to introduce the desired micro/microporosity are implemented; 2) the inherently lower modulus of most hydrogels relative to other types of biomaterials can cause challenges with stabilizing the macro or micro-porous structures or complex geometries typically sought to mimic the morphology of native extracellular matrix; and 3) the inherent cell repellency of most hydrogels (in particular actively cell-repellent/anti-fibrotic hydrogels such as poly (ethylene glycol) or zwitterionic hydrogels widely applied in tissue engineering contexts (Bai et al., 2014; Bernhard et al., 2017)) limits the degree of cell adhesion that is typically achieved. As such, to design effective hydrogel-based tissue engineering scaffolds, their chemical properties (e.g., degradation, crosslinking), physical properties (e.g., biomechanics, porosity, diffusion) and biological properties (e.g., cell type, growth factor and bioactive cues) must all be rationally designed.

**FIGURE 1:**
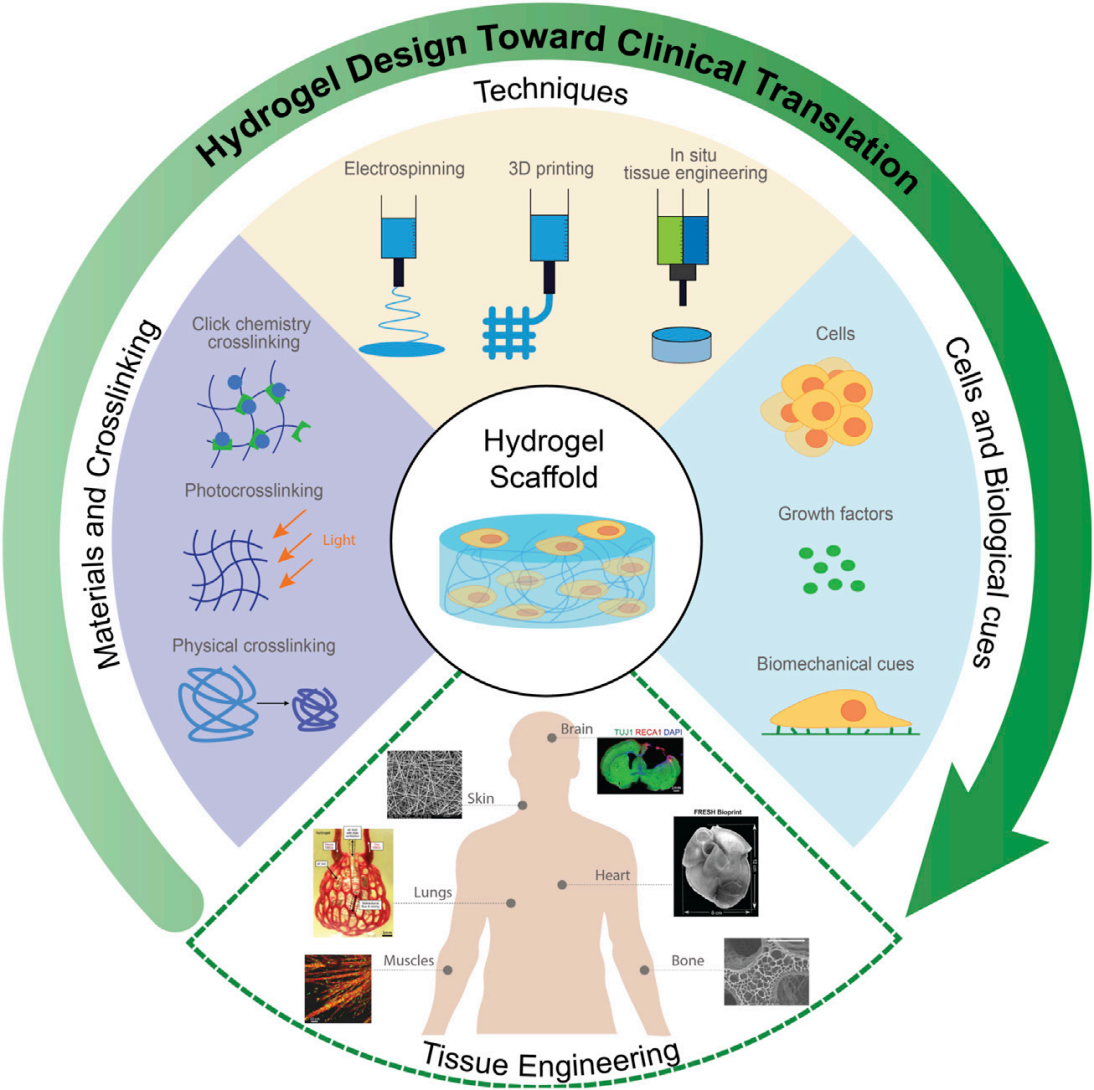
Design of functional hydrogels for tissue engineering. Selected constituent images reproduced with permission from references (Jin et al., 2011; Fuoco et al., 2014; Fang et al., 2016; Grigoryan et al., 2019; Mirdamadi et al., 2020; Li et al., 2021).

Although multiple biomaterials and techniques such as combining natural and synthetic polymers to improve cell compatibility (Place et al., 2009), incorporating biomolecules or functional groups to improve cell adhesion (Zhu, 2010), or using advanced techniques to incorporate stable micro/nanostructures in scaffolds (De France et al., 2018) have been applied to design hydrogel-based scaffolds for specific cell types, the translation of hydrogels for clinical use remains a challenge (Bhatia, 2012; Caló and Khutoryanskiy, 2015); with only a few hydrogels (e.g., Apligraf^®^, AlloDerm^®^, and Juvéderm^®^) have been approved for use in the clinic (Gaharwar et al., 2020; Mandal et al., 2020). In this review, we will focus on the design and fabrication of hydrogels for tissue engineering with particular attention to their clinical translation, including recent advances in chemistry and fabrication approaches to better mimic the extracellular matrix of soft tissues. In particular, we emphasize strategies for manufacturing tissue engineering hydrogels in which both macroporous scaffold fabrication and cell loading can be conducted in a single manufacturing step – electrospinning, 3D printing, and *in situ* tissue engineering (Figure 1). We suggest that combining such integrated fabrication approaches with the lessons learned from previously successful translational experiences with other hydrogels represents a promising strategy to accelerate the implementation of hydrogels for tissue engineering in the clinic.

## 2 Biomaterials for Hydrogel Preparation

A key consideration in the design of functional hydrogels for tissue engineering is their ability to closely mimic the native extracellular matrix (ECM) of the targeted tissues (Tibbitt and Anseth, 2009; Tsang et al., 2010). The ECM plays an integral role in maintaining tissue homeostasis by regulating cell function, tissue architecture, and storing growth factors that regulate cell adhesion and interactions (del Bakhshayesh et al., 2019), thus serving as the key signaling strategy for cell differentiation, proliferation and migration (Unal and West, 2020). As such, many examples of the use of hydrogels in tissue engineering have applied native or modified ECM materials (e.g., collagen, gelatin, fibrin) that contain specific peptide sequences (e.g., RGD, YIGSR, and others) that can interact with cell surface receptors (e.g., integrins) to promote cell adhesion and tissue growth without additional functionalization (Lei et al., 2011). However, native ECM materials also contain other types of binding domains (e.g., immunoglobulin-like adhesion molecules) that can elicit other types of biological responses that can result in poor tissue growth or undesirable side-effects (e.g., inflammation or fibrosis), particularly if the components are in some way denatured during processing (Chen and Hunt, 2007; Unal and West, 2020). In contrast, synthetic hydrogels (e.g., poly (ethylene glycol)) minimize non-specific protein adsorption and thus immune/inflammatory responses but typically have poor cell adhesion to the hydrogel scaffold (Li et al., 2012). While grafting small adhesive peptides, commonly Arg-Gly-Asp (RGD) peptides, can in part overcome this challenge, such modifications represent an additional synthetic and purification steps that can increase the cost of material and complicate the regulatory approval process (Zhu, 2010).

The degradation and clearance rate of the polymers selected is also critical for promoting tissue growth in most tissue engineering approaches (Unal and West, 2020). As cells form functional tissues, hydrogel scaffolds are typically designed to degrade and clear from the body with minimal impact to surrounding tissues and organs. Native and modified ECM proteins like collagen, elastin, fibrin and hyaluronic acid (HA) are susceptible to enzyme-mediated degradation and are metabolized into biocompatible small molecules (Smeets et al., 2014). In contrast, most synthetic hydrogels are based on polymers with carbon-carbon backbones that cannot be metabolized, thus requiring the incorporation of hydrolytically labile segments or enzyme-sensitive linkages (e.g., peptide binding domains for native enzymes) to enable controlled degradation (Zhu, 2010), typically into oligomeric by-products with molecular weights appropriate for renal clearance (<60 × 10^3^ g/mol) (Pasut and Veronese, 2007).

Finally, the compositional and morphological diversity (and thus range of accessible physicochemical properties) of a given hydrogel in relation to its target application should be considered in choosing the correct hydrogel material. The exceptional control that modern polymer chemistry techniques can impart on both the chemistry and the molecular structure of a synthetic polymer can enable precise tailoring of the physiochemical and mechanical properties of synthetic hydrogels in a way that is challenging to reproduce with naturally-derived polymers that typically have broad molecular weight distributions and complex variable compositions (Place et al., 2009). Synthetic polymers also offer higher reproducibility and significantly less potential for batch contamination given that they avoid the need for the complex purification protocols required to isolate natural polymers from their animal or plant source (Hutmacher, 2010; Unal and West, 2020); in particular, naturally derived and modified ECM components pose significant challenges in terms of the removal of contaminants such as proteins (e.g., non-tissue relevant structural proteins, immunity-triggering xenograft proteins), polyphenolics, endotoxins, RNA, and DNA which can trigger undesirable biological responses and thus significantly complicate practical clinical use (Gilpin and Yang, 2017; Montalbano et al., 2018).

**FIGURE 2:**
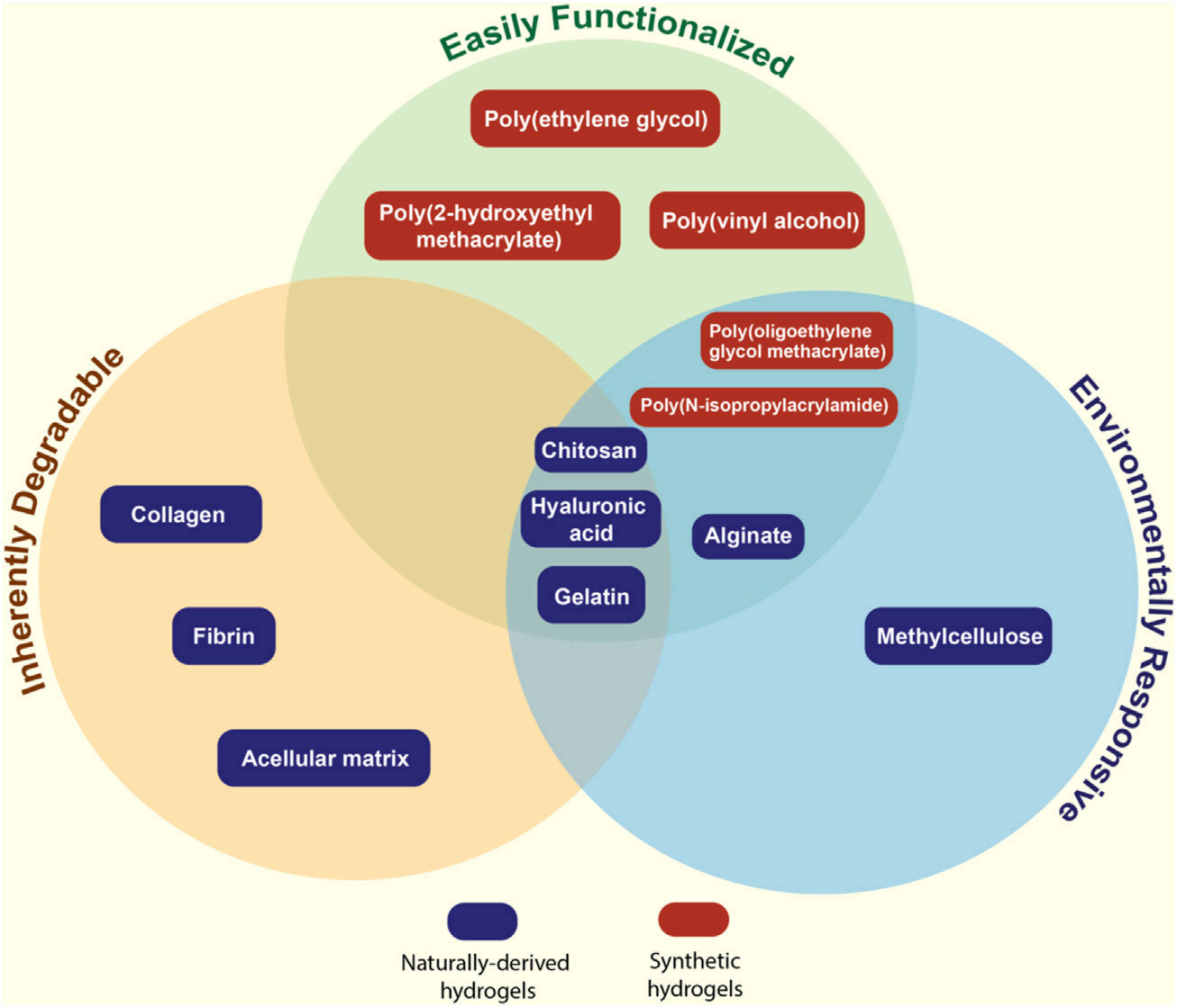
Venn diagram describing the key properties of natural and synthetic polymers most commonly used for fabricating hydrogel-based tissue scaffolds.

Balancing these competing considerations makes the selection of the type of polymer(s) (natural or synthetic) and the specific polymer(s) within one of those two groups to design a hydrogel-based tissue engineering scaffold complex (Figure 2). In the following sections, we will outline the major types of natural and synthetic polymers used to form hydrogels, the major strategies available to crosslink those polymers to form hydrogels, and the relative advantages and disadvantages of each in the context of practical tissue engineering applications.

### 2.1 Naturally-Derived Hydrogels

#### 2.1.1 Collagen

Collagen is a principal component of the ECM typically isolated from mammalian bone, cartilage, skin, tendons, and ligaments (Antoine et al., 2015). Collagen hydrogels, commonly based on type I collagen that constitutes the large majority of the total collagen found in the body, are typically formed through hydrogen bond-driven self-assembly into fibrils under physiological temperature irrespective of pH (Ferreira et al., 2012), although other approaches such as crosslinking with small molecules (e.g., glutaraldehyde, genipin, or EDC/NHS (1-ethyl-3-(3-dimethylaminopropyl) carbodiimide/N-hydroxysuccinimide)) or enzyme-induced crosslinking (e.g., microbial transglutaminase (MTG)) have also been reported (Garcia et al., 2007; Adamiak and Sionkowska, 2020). Like many naturally derived hydrogels, the amino acid sequence of collagen is easily recognized by host cells and can be degraded *in vivo* by naturally occurring collagenase (Seibel et al., 2006). Naturally occurring binding domains on collagen can promote cell adhesion and cell-cell interactions, allowing encapsulated cells to develop into functional tissues (Glowacki and Mizuno, 2008). Furthermore, the larger pore sizes typically observed following the self-assembly of collagen provide physical space for cell proliferation, although also making the gels susceptible to relatively rapid degradation *in vivo* that can limit accessible culturing times before native cell ECM production must take replace the structural properties of the delivered scaffold (Song et al., 2006; Ferreira et al., 2012). The production of collagen hydrogels is however associated with high manufacturing costs stemming from the time-consuming purification and isolation procedures required for collagen isolation (Nagai et al., 2004). Despite its primary role of structural support in the ECM, collagen-based hydrogels also typically have poor mechanical strength in comparison to many synthetic hydrogels (Antoine et al., 2015; Montalbano et al., 2018). Mixing collagen with other biomaterials (most typically other natural polymers such as alginate, gelatin, or fibrin) can in part improve such properties. For example, Montalbano et al. reported that collagen concentrations of 2.5 w/v% in collagen-alginate-fibrin thermoresponsive hydrogels promoted increased fibril homogeneity, faster gelation times, greater stability, and sustained cell morphology relative to collagen-only hydrogels, although care must be taken to maintain high nutrient transfer and metabolite diffusion in denser collagen networks (Montalbano et al., 2018).

#### 2.1.2 Gelatin

Gelatin is an inexpensive and readily available material derived from denaturing the triple-helix structure of collagen into single strands (Elzoghby, 2013). Gelatin hydrogels undergo facile physical crosslinking under low temperatures and can hold significant amounts of water, favorable for nutrient transport (van den Bulcke et al., 2000). Since gelatin is a derivative of collagen, it also contains RGD binding motifs for cell adhesion and degrades into non-toxic resorbable products, with the denatured state lowering the antigenicity of gelatin compared to collagen (Elzoghby et al., 2012). However, the low stability of gelatin hydrogels at physiological temperatures in the absence of chemical crosslinks (or another type of temperature-insensitive physical crosslink) typically imparts poor mechanical strength (van den Bulcke et al., 2000). Grafting gelatin with methacrylic anhydride to create gelatin methacryloyl (commonly referred to as GelMA) that is UV photocrosslinkable offers an alternative to introduce covalent crosslinks into the gelatin scaffolds to improve their stability and has been widely applied for tissue scaffold development, although such functionalization requires additional synthetic grafting and subsequent polymerization (typically photopolymerization) steps. As with collagen, combinations of gelatin with other natural polymers are also often pursued to address the mechanical limitations of native gelatin hydrogels. For example, Shen et al. fabricated gelatin-chitosan hydrogels to recreate ideal scaffold degradation rates and pore sizes for cartilage formation, with the added gelatin enabling the reduced pore size, increased mechanical strength and increased elasticity required to grow cartilage (Shen et al., 2015).

#### 2.1.3 Fibrin

Fibrin, formed by thrombin-mediated crosslinking of fibrinogen, plays a critical role in the regulation of tissue homeostasis and wound healing (Ehrbar et al., 2007; Ahmed et al., 2008). Similar to collagen and gelatin, fibrin contains multiple integrin and cell binding domains (including the RGD sequence most commonly implicated in integrin-mediated cell adhesion) (Janmey et al., 2009) and degrades into non-toxic by-products via enzymatic (plasmin-mediated) degradation (Janmey et al., 2009); unlike other natural hydrogels, fibrin networks rapidly self-crosslink via polycondensation reactions catalyzed by thrombin to form relatively stiffer gel matrices (Janmey, 1982) with the gelation time and the mechanics of the resulting fibrin hydrogels controllable by modifying thrombin concentrations (Janmey et al., 2009). However, consistent with its role in reversible clot formation, fibrin gels typically degrade rapidly in 15 days (Bensaïd et al., 2003; Noori et al., 2017). As such, combinations of fibrin with other biomaterials (e.g., medical biodegradable aliphatic polyurethane or alginate) have been reported to prolong the residence time of the hydrogel scaffold to better match the targeted rate of tissue regeneration (Lee et al., 2005; Deepthi and Jayakumar, 2018).

#### 2.1.4 Hyaluronic Acid

Hyaluronic acid (HA) is a linear polysaccharide that is found throughout the body but particularly in connective tissues (Price et al., 2007). HA plays a critical role in tissue hydration, nutrient diffusion, proteoglycan organization and cell differentiation (Tan et al., 2009). Similar to other natural polymers derived from the ECM, HA is degraded by host enzymes - specifically, hyaluronidase which is found in serum (Smeds and Grinstaff, 2001). HA is also upregulated in tissues with high growth rates and at wound sites due to the key role of HA in promoting cell spreading and proliferation (Burdick and Prestwich, 2011). Compared to other natural materials such as collagen and gelatin, HA is easily modified to diversify its mechanism of gelation, with a range of functional groups including thiol, hydrazide, aldehyde, and tyramine groups all reported to prepare crosslinked hydrogels (Burdick and Prestwich, 2011). For example, Park et al. prepared an injectable HA hydrogel using tetrazine-modified HA (HA-Tet) and transcyclooctene-modified HA (HA-TCO) that can be crosslinked *in situ* under physiological conditions via a Diels–Alder click reaction (Park et al., 2019). The *in situ* crosslinked HA hydrogel can enable chondrogenic differentiation of encapsulated human periodontal ligament stem cells (hPLSCs) as induced by cytomodulin-2 (CM) that was also covalently linked to HA (Park et al., 2019). However, the extremely high-water binding capacity of HA can limit the concentration of the polymer that can be used to fabricate hydrogels based on the high viscosity of the precursor polymers, thus also limiting the resulting mechanics of the resulting hydrogels.

#### 2.1.5 Chitosan

Chitosan is a positively charged polysaccharide formed through the deacylation of chitin, the main structural component of crustacean exoskeletons (Islam et al., 2020). Although chitosan is not found natively in human ECM, the somewhat unique cationic charge of chitosan among carbohydrates can promote cell adhesion via electrostatic interactions, avoiding the common need for incorporating specific peptide motif cell binding sites into ECM-mimicking hydrogels. Chitosan hydrogels can be formed via physical crosslinking (typically hydrogen bonding, although such hydrogels are weak and can be highly pH-sensitive (Nicodemus and Bryant, 2008)), ionic complexation (e.g., with polyaspartic acid sodium salt or tripolyphosphate), or covalent crosslinking (e.g., with glutaraldehyde or formaldehyde), although the covalent crosslinkers typically used can pose significant toxicity challenges if residual crosslinker is not fully removed from the gel after crosslinking is complete (Hennink and van Nostrum, 2012; Cho et al., 2016). Chitosan is non-toxic (with proper purification), can have low immunogenicity (Nicodemus and Bryant, 2008), can adhere strongly to mucosal surfaces, and has some inherent anti-bacterial properties that may be beneficial to prevent post-implantation infections (Samprasit et al., 2015; Karava et al., 2020). However, native chitosan is insoluble at neutral pH (thus requiring the use of acidic solutions to fabricate hydrogels that can pose cell toxicity challenges). Chitosan is also not inherently biodegradable, although oxidative degradation of the glycosidic bonds can occur over time to break chitosan back down to oligomeric sugars (Einbu et al., 2007). Modified chitosan derivatives such as carboxymethyl chitosan can overcome the solubility problem but can partially dilute some of the benefits of chitosan in terms of cell adhesion or anti-bacterial properties. While chitosan has been used in a range of tissue engineering applications, these hydrogels have notably been found to promote bone formation by increasing alkaline phosphatase activity and calcium deposition in osteogenic mediums (Wang and Stegemann, 2010).

#### 2.1.6 Alginate

Alginate is a linear anionic polysaccharide isolated from brown algae that can rapidly form hydrogels upon exposure to divalent cations (Augst et al., 2006; Bidarra et al., 2014). Calcium is the most common crosslinking ion and facilitates relatively strong alginate gelation through the formation of an “egg crate” structure in which four alginate residues interact with a single calcium, although other alkali earth metal ions (in particular barium) can also be used as crosslinkers to modify the stability of the ionic crosslink in different environments (Bidarra et al., 2014; Matyash et al., 2014). Although ionic crosslinking mechanism is simple and highly cytocompatible, solutes present in the microenvironment can strongly influence alginate crosslinks and can result in poorly controlled degradation through ion exchange with the high concentration of monovalent ions in the physiological environment (Neves et al., 2020). Alginate also cannot inherently promote cell adhesion, with co-formulation with other ECM components that can be physically entrapped within the rapidly forming alginate-calcium hydrogel (e.g., collagen) and/or cell adhesion peptides grafted to alginate both found to improve adhesion (Moxon et al., 2019). Furthermore, while degradation of the hydrogel is facile via ion exchange, clearance of the alginate polymer itself can be slow if the molecular weight exceeds the renal cut-off, with only oxidation available to degrade the polymer itself *in vivo* (Lueckgen et al., 2019). Chemical modifications on the hydroxyl groups or carboxyl groups of alginate have been developed to improve the physiochemical and mechanical properties of alginate hydrogels (Neves et al., 2020), with methacrylation being the most popular method to enable the fabrication of dual ionic (calcium-induced gelation)/covalent (photogelation of methacrylate groups) crosslinked hydrogels that can achieve significantly higher moduli than either crosslinking approach can achieve alone (Samorezov et al., 2015).

#### 2.1.7 Methylcellulose

Methylcellulose (MC) is a non-toxic, degradable, and thermoresponsive polymer derived from cellulose (Adamiak and Sionkowska, 2020). Given that the sol-gel transition temperature of MC is close to the body temperature, MC has been used to prepare thermoresponsive hydrogels that have been widely applied in tissue engineering, including *in situ* gelling systems for cell and biomolecule delivery, bioprinting inks, and surface modifications for cell adhesion (Adamiak and Sionkowska, 2020). The lower critical solution temperature (LCST) of MC can be adjusted by either changing the properties of MC (e.g., concentrations, degree of substitution, modifications) or the external environment (e.g., anions, solvent, electromagnetic fields) (Adamiak and Sionkowska, 2020). However, purely physically crosslinked MC hydrogels cannot provide a long-term mechanical support for cell encapsulation, requiring the co-formulation with other materials or chemical modifications to improve the mechanical properties and/or longevity of the MC-based scaffolds. For example, Shin et al. prepared a tyramine-modified MC hydrogel with improved mechanical properties by combining MC-driven thermally-induced crosslinking with photocrosslinking and demonstrated the utility of the material for 3D bioprinting (Shin et al., 2020), while the Shoichet group developed an injectable hydrogel blend composed of hyaluronan and methylcellulose (HA-MC) in which HA was added to improve the mechanics of the gel and promote shear thinning to aid in injectability at higher polymer concentrations without compromising the thermogelation capacity of the MC component (Tam et al., 2012; Ho et al., 2019).

### 2.2 Synthetic Hydrogels

#### 2.2.1 Poly (Ethylene Glycol) and Derivatives

Poly (ethylene glycol) (PEG) is a synthetic hydrophilic polymer used for a variety of biomedical applications due to its low cytotoxicity, non-immunogenicity and non-specific protein adhesion properties (LEE et al., 1995; Zhu, 2010). PEG-based hydrogels are typically formed by the free radical polymerization of bifunctional PEG diacrylate (PEGDA) or PEG dimethacrylate (PEGDMA) macromonomers, with the average pore size of the gel directly controllable based on the average chain length of the PEG chains between the crosslinker groups (Reid et al., 2015). Multi-arm PEG acrylates or end-functionalized PEG precursors (e.g., those crosslinkable by click chemistry with complementary functional groups) have also been formed that offer the potential to form ideal hydrogels with highly defined pore structures (Bakaic et al., 2015). As such, the degree of hydrogel structure control achievable with PEG is very high. However, the high cell repellency of hydrogels represents a drawback of this material for practical tissue engineering applications, typically requiring the incorporation of other entities such as grafted cell adhesion peptides, hydrophobic block copolymers (e.g., PEG-*block-*poly (ε-caprolactone), PEG-PCL) or thermoresponsive block copolymers (PEG-*block-*poly (propylene oxide), PEG-PPO); the latter two approaches also enhance the mechanics of the hydrogel via a dual covalent bonding/hydrophobic interaction crosslinking mechanism) (Zhu, 2010; Bakaic et al., 2015). The choice of crosslinking agent(s) can directly influence hydrogel degradation, swelling, porosity, and mechanical strength as desired for a specific tissue engineering application. For example, Liu et al. mixed non-degradable PEG-dithiol and degradable PEG-metalloproteinase crosslinkers in various ratios with 4-armed PEG-MAL to create enzymatically degradable PEG hydrogels with tunable degradation rates, pore size and mechanical strengths relevant to promote adipocyte and osteoblast differentiation (Liu et al., 2020). However, unless degradation is directly incorporated into the network (as in the Liu et al. example above), PEG is not inherently degradable aside from slow oxidation that may occur at the ether linkages in the main PEG chain. In addition, increasing concern about the development of PEG antibodies (estimated to exist in up to 89% of the patients (Sundy et al., 2011; Zhang et al., 2016) due to environmental exposure to PEG in personal care products and laxatives, among others) may result in an unanticipated immune response to a PEG-based material (Garay et al., 2012; Zhang et al., 2016). PEO-based block copolymers such as poly (ethylene oxide)-b-poly (propylene oxide)-b-poly (ethylene oxide) (PEO-PPO-PEO), commercially known as poloxamers, Pluronics®, Synperonics® or Lutrol^®^, are particularly notable given their potential to undergo thermal gelation at body temperature without the need for any external chemicals or post-treatment (Russo and Villa, 2019; Zarrintaj et al., 2020). The *in situ* gelation of poloxamers has been leveraged to create injectable hydrogels for cell delivery and tissue regeneration (Russo and Villa, 2019). However, the micelle-based gelation mechanism typically results in weaker hydrogels that can disassemble relatively quickly *in vivo*. Hybrid materials that combine poloxamers with other gelling polymers offer promise to address this challenge, often using the poloxamer to rapidly immobilize the hydrogel at the site of injection to allow further fixation to occur. For example, Suntornnond et al. mixed Pluronic F127 (poloxamer 407) with GelMA to prepare a printable hydrogel ink for fabricating vasculature-like structures (Suntornnond et al., 2017). The micelle formed by Pluronic improved the printability of the composite hydrogel ink at a wide range of temperatures while GelMA can be further crosslinked by UV light to improve the mechanical stability of printed structures (Suntornnond et al., 2017). The surfactant-like structure of poloxamers can however introduce challenges with preserving high cell viability within the scaffold, causing changes in the lipidic profile, or inducing renal toxicity (Dumortier et al., 2006) that may limit the translational potential of poloxamers.

#### 2.2.2 Poly (Vinyl Alcohol)

Poly (vinyl alcohol) (PVA) has attracted attention for its high-water retention and superior mechanical strength to PEG, enabling its use in applications requiring stiffer scaffolds such as the replacement of articular cartilage (Schmedlen et al., 2002). PVA hydrogels are typically formed via a simple repeated freeze-thawing process, resulting in the formation of strong hydrogen bonded crosslinked networks with tunable pore sizes based on the rate and frequency of freezing used. PVA hydrogels can also be prepared using freeze-drying techniques by taking advantage of the same strong hydrogen bonding networks (Vrana et al., 2009); using either technique, elastic, non-toxic and stable hydrogels can be formed at room temperature. However, neither process is readily adaptable to injectable use like many other polymers described, which may limit the use of PVA to surgical implantation rather than injectable scaffold formation. PVA is a non-degradable polymer under physiological environment and does not inherently promote cell adhesion, which limits the applications in tissue regeneration. Moreover, the hydrogen bonding-based crosslinking strategy of PVA increases the difficulties of chemical modifications with other polymers that can address these drawbacks. The formation of composite PVA hydrogels by physically encapsulating natural ECM components such as collagen or gelatin (Wu et al., 2018) or 3 (Lim et al., 2013) have both been pursued to avoid this problem; in the latter case, a tyramine-functionalized poly (vinyl alcohol) (PVA-Tyr) polymer was synthesized that can be crosslinked via photopolymerization and degraded via hydrolysis of the ester bond linking the Tyr groups with PVA, with gelatin also incorporated via interactions of the tyrosine fractions of gelatin and tyramine groups of PVA-Tyr to enable improve cell adhesion (Lim et al., 2013).

#### 2.2.3 Poly (N-Isopropylacrylamide)

Poly (*N*-isopropylacrylamide) (PNIPAAm) is an amphiphilic temperature-responsive smart polymer typically fabricated via free radical polymerization. PNIPAM has a lower critical solution temperature (LCST) of ∼32°C and therefore can form a hydrogel at physiological temperatures by thermally-driven self-assembly (Haq et al., 2017); however, the highly dehydrated state of the self-assembled polymers at 37°C has motivated covalent crosslinking of PNIPAM and/or the co-incorporation of more water-binding components into the hydrogel (e.g., by copolymerization of more hydrophilic comonomers or physical encapsulation of hygroscopic polymers (Jin et al., 2008; Ding et al., 2020)) to maintain higher water contents under physiological conditions. The more hydrophobic character of PNIPAM at body temperature can promote cell adhesion via hydrophobic interactions despite the lack of specific cell binding domains (Ashraf et al., 2016), although the collapse of the gel can result in relatively low pore sizes that may hinder cell growth and signaling (Atoufi et al., 2019). The main drawback of PNIPAM is the high toxicity of the NIPAM monomer (Joseph et al., 2021), which requires extensive purification of the hydrogel prior to practical use *in vivo* and has posed translational challenges with other PNIPAM-based technologies (Capella et al., 2019). PNIPAM is also not inherently degradable *in vivo*, although crosslinking PNIPAM oligomers that are renally clearable via hydrolytically labile bonds can address this degradation/clearance challenge (Patenaude and Hoare, 2012).

#### 2.2.4 Poly (Oligoethylene Glycol Methacrylate)

Poly (oligoethylene glycol methacrylate) (POEGMA) has a methacrylate-based backbone (making it polymerizable via free radical polymerization) and PEG-based side chains. By tuning the length of the PEG side chains, the polymer properties can be switched from being temperature-responsive (*n* = 2–3 ethylene oxide side chains) to being highly protein-repellent (*n* > 5–6 ethylene oxide repeat units) (Patenaude et al., 2014; Smeets et al., 2014); coupling this benefit with the capacity to include any type or number of functional group(s) in the polymer via simple free radical copolymerization and the noted lower (or absent) immune response to the material (Bakaic et al., 2015; Chen et al., 2021), POEGMA avoids many of the challenges of PEG while maintaining its beneficial non-toxic, non-immunogenic, and protein repellent properties. We have demonstrated that incorporating aldehyde and hydrazide moieties into POEGMA-based polymers enables the formation of injectable *in situ-*gelling and hydrolytically-labile hydrazone crosslinked hydrogels with highly tunable pore size, gelation time and mechanical strength without inducing any significant cytotoxic effects *in vitro* or *in vivo*; keeping the precursor polymer molecular weight < 40 kDa maintains the potential for clearance upon hydrolysis of the hydrazone crosslink even in the context of the non-degradable C-C backbone (Smeets et al., 2014; Bakaic et al., 2015). POEGMA offers an attractive alternative to PNIPAM and PEG because of its non-toxic degradation products and facile functionalizability, respectively (Smeets et al., 2014; Bakaic et al., 2015). *In situ* gelling thermosensitive POEGMA hydrogels that can undergo phase transitions at a range of physiologically relevant temperatures can be obtained by mixing di(ethylene glycol) methyl ether methacrylate (M(EO)_2_MA) with longer chain (*n* = 7–8 ethylene oxide repeat units) OEGMA monomers, with the transition temperature varying linearly with the mole percentage of each monomer (Lutz et al., 2006) while preserving a relatively sharp temperature response (unlike with PNIPAM, in which transitions become much broader when hydrophilic comonomers are incorporated) (Smeets et al., 2014; Xu et al., 2021). In addition, although POEGMA does not contain inherent binding cell binding domains, the thermo-reversible natural of POEGMA smart gels enables cell adhesion at temperatures above the volume phase transition temperature of the hydrogel (Xu et al., 2021). For example, Smeets et al. demonstrated good cell adhesion, mild inflammatory responses, and good stability (over several weeks) using hydrazone-crosslinked POEGMA hydrogels *in vivo* (Smeets et al., 2014).

#### 2.2.5 Poly (2-Hydroxyethyl Methacrylate)

Poly (2-hydroxyethyl methacrylate) (PHEMA) hydrogels were first introduced in the 1960s for use in contact lenses and have since been widely explored in tissue engineering given that they have many of the same advantages as PVA and PEG previously described (Xinming et al., 2008; Zare et al., 2021). However, unlike PEG, it can be freely copolymerized with other comonomers (making functionalization easier) and, unlike PVA, it is easily crosslinkable via a range of different strategies (making its degradability more controllable). Creating scaffolds that combine PHEMA with other natural (e.g., HA (Huang et al., 2013) or dextran (Meyvis et al., 2000)) and synthetic polymers can optimize the mechanical strength, non-immunogenicity, and physical properties of PHEMA gels to match those of living tissues (Bach et al., 2012). Similar to other synthetic hydrogels, PHEMA hydrogels are not inherently degradable in physiological conditions, requiring the incorporation of hydrolytic or enzyme-degradable segments, although such copolymerization is relatively facile with free radical copolymerization or derivatization of the alcohol side groups. (Meyvis et al., 2000). For example, Dragusin et al. prepared gelatin-pHEMA hydrogel scaffolds by photocrosslinking HEMA with methacrylamide-modified gelatin, with the incorporation of gelation improving the swelling properties, enhancing cell adhesion, and enabling enzymatic control over gel degradation (Dragusin et al., 2012). PHEMA hydrogels have also been noted to undergo calcification after long-term implantation, a potential benefit for bone tissue engineering but a potentially negative consideration for regenerating other soft tissues (Vijayasekaran et al., 2000).

### 2.3 Crosslinking Methods

Once the backbone polymer is chosen, the method of crosslinking the polymer chains together must be judiciously selected. Physical crosslinking and chemical crosslinking strategies are available, each with their own advantages and drawbacks in the context of tissue engineering. Compared to physical crosslinking, chemical crosslinking strategies typically result in linkages with more controllable degradation profiles and better mechanical properties; however, chemical crosslinking typically requires some kind of chemical derivatization of the backbone polymer and/or additional synthetic steps that may not be fully compatible with cells, unlike most physical crosslinking processes (Hennink and van Nostrum, 2012; Hu et al., 2019). *In situ* gelling in which the hydrogel gels upon injection *in vivo* via either physical crosslinking (i.e., ionic crosslinking, thermoresponsive phase transitions) or chemical crosslinking (i.e., the application of visible light/UV irradiation or click chemistry) offers particular promise for preparing injectable hydrogels for cell delivery in that such hydrogels avoid the need for surgical implantation, facilitating their practical clinical use (van Tomme et al., 2008). Herein, we will only briefly summarize the major categories of crosslinking approaches used for the fabrication of tissue engineering scaffolds; for more details on the options, we refer the interested reader to more in-depth reviews on hydrogel crosslinking strategies (Hennink and van Nostrum, 2012; Hu et al., 2019; Mueller et al., 2022). Note that, while the most common method(s) used to crosslink each polymer previously discussed were described in Sections 2.1 and 2.2, in principle any chemical crosslinking approach could be pursued with any of polymer by exploiting either native functional groups (e.g., all naturally-sourced polymers) or by introducing functional groups via, e.g., copolymerization (e.g., any synthetic polymer described); in principle, subsequent conversion of those functional groups to another functional group suitable for a specific crosslinking reaction could be conducted to crosslink any given polymer with any specific type of linkage. However, a few physical interactions (e.g., the egg crate Ca^2+^/alginate interaction or the freeze-thaw gelation of PVA) tend to be more specific to particular polymer types.

#### 2.3.1 Physical Crosslinking

Networks of physically crosslinked hydrogels are generally formed through ionic/electrostatic interactions, hydrogen bonding, metal-ligand interactions, polymer chain entanglements, hydrophobic interactions, and/or host-guest interactions between polymers or between polymers and small molecule crosslinkers (Hennink and van Nostrum, 2012; Hu et al., 2019). Due to the absence of chemical crosslinkers and solvents, physical crosslinking can enable the preparation of cell compatible hydrogels under a mild environment (e.g., at room temperature); moreover, physical crosslinking typically does not require the functionalization of the gel precursor polymers and is often highly reversible, both beneficial to practical clinical translation (Hu et al., 2019). Ionic crosslinking interactions by which polysaccharides such as alginate (Bidarra et al., 2014), chitosan (Moura et al., 2011), and cellulose derivatives (Chang and Zhang, 2011; Du et al., 2019), can be crosslinked by cations (e.g., Ca^2+^, Mg^2+^, Fe^3+^) have been particularly widely leveraged to prepare hydrogels for tissue engineering given the high cell compatibility of the divalent cation crosslinkers, although as previously noted ion exchange with the ions in the physiological environment can make it challenging to control the degradation of the hydrogels (Anamizu and Tabata, 2019). Hydrogen bonding interactions, particularly following freeze-thaw processes that help enhance chain alignment and thus hydrogen bonding, have also been widely used, particularly using poly (vinyl alcohol) (PVA) as the main polymer and optionally including other polymers and/or nanoparticles (e.g., cellulose nanocrystals (CNCs)) with hydrogen bonding groups (Lin et al., 2020). However, given the highly aqueous environment of a hydrogel, only strongly hydrogen bonding precursors (such as PVA) will yield hydrogels with reasonable stability following implantation. Hydrophobic interactions, particularly those driven by thermoresponsive polymers that transition from being soluble at lower temperature to self-associative at higher temperature (e.g., methylcellulose (MC) and Pluronic-based materials) can enable gelation while also providing more hydrophobic adhesion sites for cells, although the degradation of such hydrogels can also be challenging to control (Park et al., 2017). Supramolecular host-guest interactions, often involving β-cyclodextrin (βCD) interactions with guest compounds such as poly (ethylene oxide) and adamantane, have also been widely used given their highly effective shear thinning/self-healing properties and facile tuning of matrix stiffness, although the materials available to form such hydrogels are limited to those that can form sufficiently strong inclusion complexes (Hörning et al., 2017; Liu et al., 2018). As such, in general, physical crosslinking can be highly beneficial for tissue engineering applications but can limit the types of materials that can be used and/or introduce challenges with tuning the degradation rate of the gel to that of the cell proliferation process.

#### 2.3.2 Chemical Crosslinking

Chemical crosslinking for the fabrication of tissue engineering matrices can typically be categorized into one of two main strategies: photocrosslinking and click chemistry. In photocrosslinking, light (e.g., most commonly UV light but in some cases visible light) is used to initiate the polymerization of unsaturated vinyl, acrylate, methacrylate or allylic groups conjugated to the gel precursor polymers, typically aided by water-soluble photoinitiators (e.g., Irgacure 2959) to promote free radical generation under lower intensities of light than would be required to form a gel via only hydrogen abstraction from the precursor polymers (Stephens-Altus et al., 2011). While photocrosslinking has been used successfully for a range of different cell types, the potential risks caused by UV radiation (e.g., DNA damage, aging, etc.) can still be concerns, particularly for less robust cell lines. Visible-light-initiated crosslinking such as ruthenium-catalyzed photocrosslinking under blue light with a wavelength of 458 nm can avoid these issues but can complicate the chemistry involved in the gelation process (Bjork et al., 2011; Fernandes-Cunha et al., 2017). Of note, *in situ* photopolymerization in which the scaffold is irradiated as it is administered *in vivo* has attracted increasing interest and has been used successfully for the repair of corneal wounds, *in situ* cartilage regeneration, and other applications (Smeds and Grinstaff, 2001; Burdick and Anseth, 2002; Leach et al., 2004).

Click chemistry, a range of reactions that can occur rapidly and spontaneously under physiological conditions without producing toxic by-products (i.e., no by-products or water) has also attracted significant increasing attention given that no additional crosslinkers/initiators/catalysts are required to prepare the hydrogels, no post-treatment is necessary, and (if delivered using a double barrel syringe and/or a static mixer) hydrogels can be administered directly into the body by simple injection to avoid the need for surgical implantation (Crescenzi et al., 2007). A range of chemistries including Diels–Alder reactions, Michael additions, oxime formation, Schiff base formation, disulfide formation, boronate ester formation and (with the addition of a low-intensity UV stimulus) thiol-ene reactions have been reported used to prepare multiple hydrogels under physiological conditions (Mueller et al., 2022).

A range of other chemical crosslinking methods based on di/multi-functional small molecule crosslinkers has been reported to prepare hydrogels (Kabiri et al., 2003; Hennink and van Nostrum, 2012; Hu et al., 2019). However, the inherent tissue toxicity many small molecule crosslinkers (in particular aldehydes such as glutaraldehyde and similar molecules) offers a potential risk for clinic use that is significantly mitigated by synthesizing polymeric analogues which tend to exhibit significantly lower toxicity. One possible exception to this rule is genipin, a natural aglycone extracted from plants that can crosslink amines (analogous to di/polyaldehydes like glutaraldehyde) while inducing significantly lower cytotoxicity (Yu et al., 2021); however, the strong purple color of this crosslinker may be non-ideal in some tissue engineering applications.

#### 2.3.3 Other Crosslinking Strategies

Enzymatic approaches can also be used to crosslink hydrogels, particularly beneficial in terms of leveraging naturally-occurring crosslinking strategies (e.g., in clotting) to create hydrogels with low inherent immunogenicity (Moreira Teixeira et al., 2012). Enzymatic reactions also typically occur under physiological conditions to enable the formation of *in situ* gelling hydrogels under physiological conditions in the presence of cells (Sperinde and Griffith, 1997; Jin et al., 2010; Moreira Teixeira et al., 2012). For example, Wang et al. reported an injectable gelatin-hydroxyphenylpropionic acid (Gtn-HPA) hydrogel that crosslinked by hydrogen peroxide (H_2_O_2_) and horseradish peroxidase (HRP) in which the stiffness of hydrogel was directly tunable by adjusting the concentrations of H_2_O_2_ and Gtn-HPA to optimize the proliferation of chondrocytes (Wang et al., 2014). However, the fast degradation and typically poor mechanical properties of enzymatically crosslinked hydrogels can limit their practical applications for tissue regeneration (Moreira Teixeira et al., 2012).

As another alternative, instead of using physical, chemical, or enzymatic processes to crosslink individual polymers together that were not previously networked together, ECM-mimetic hydrogel scaffolds can also be created by decellularizing native tissues. Decellularization refers to the removal of cells and other potential components that may introduce an immune/inflammatory response (e.g., cellular DNA, lipopolysaccharides) from native tissues using methods that do not disrupt the strong physical and/or chemical interactions in the native ECM (Saldin et al., 2017; Sackett et al., 2018), methods that may include chemical stimuli (i.e., surfactants or acids/bases to degrade cell membranes), enzymatic stimuli (e.g., trypsin, dispase, nucleases and phospholipase A2), physical stimuli (e.g., high pressure, supercritical carbon dioxide, or freeze-thaw cycles), or combinations thereof (Gilpin and Yang, 2017). The benefit of decellularization is that the internal structure and chemistry (including crosslinking) of the native ECM (ideally from the same tissue targeted for regeneration) is directly reproduced to grow the new tissue without the need to perform any additional crosslinking or structuring step. In addition, and critically given the poor availability of human donor tissues, non-human sources may still be useful for acquiring the implant scaffolds given the washing procedures used to remove potential immunogenic components (Fernández-Pérez and Ahearne, 2019). However, the challenges inherent in tissue sourcing and the extensive purification processes required to make decellularized scaffolds as well as the potential denaturation of some ECM components upon processing do offer some drawbacks to this strategy that can be avoided with the use of other scaffold building blocks.

## 3 Emerging Fabrication Techniques for Hydrogel-Based Tissue Scaffolds

**FIGURE 3:**
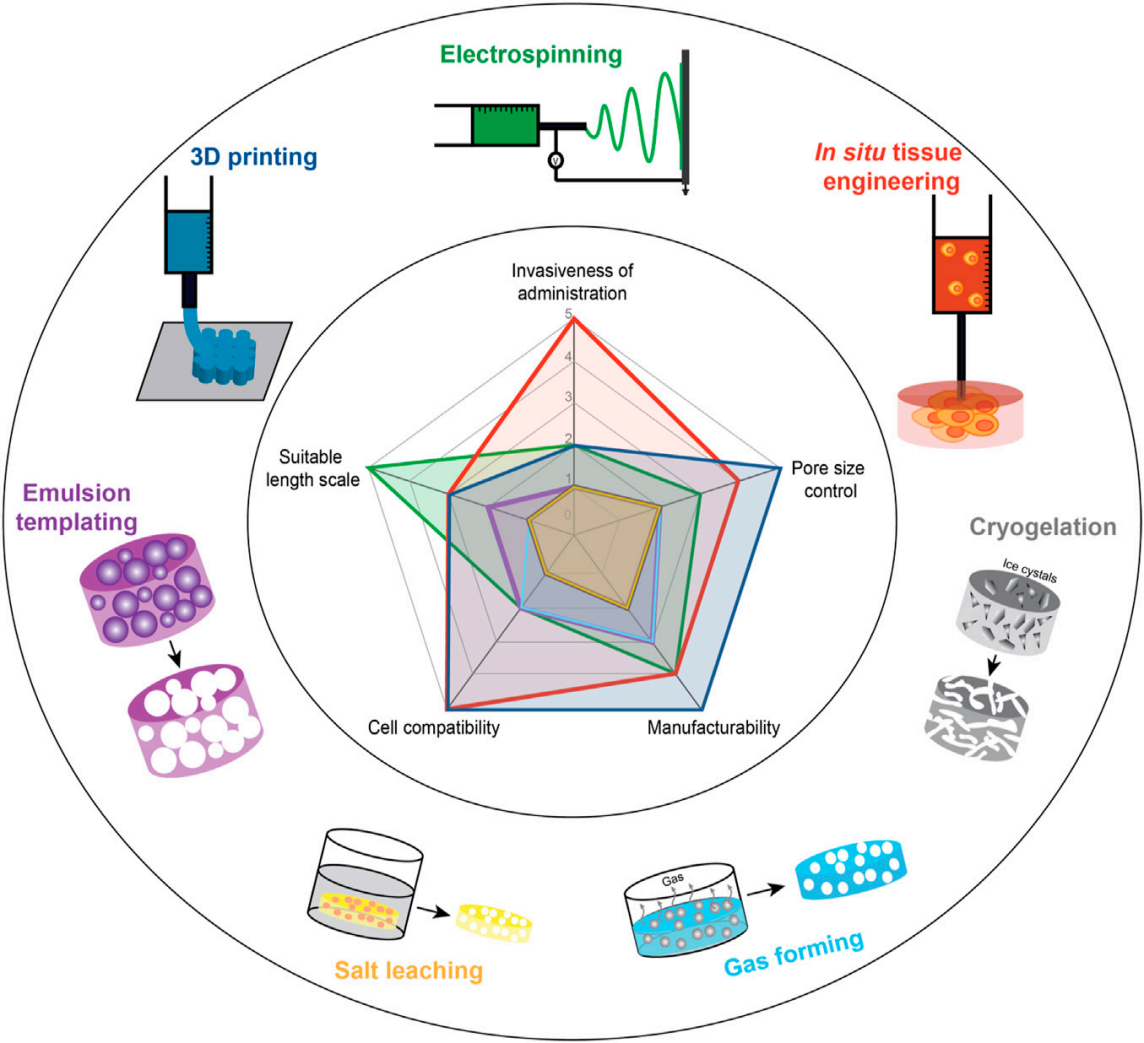
Schematic of techniques to fabricate hydrogel-based macroporous scaffolds for tissue engineering. (Inset) Spider plot of the relative advantages of different macroporous scaffold formation techniques (scale 1–5: 1 = least advantageous, 5 = most advantageous) Note that the cryogelation plot overlaps with the salt leaching plot such that it is not clearly visible in the graph.

To prepare functional tissue constructs using the materials and crosslinking approaches described, the micro- or nano- structure of the hydrogel scaffold must also be carefully controlled given the critical role that the ECM structure plays in regulating cell adhesion, migration, and proliferation as well as the degradation rate of the scaffold (Seliktar, 2012; Schulte et al., 2013; de France et al., 2018). For example, collagen, elastin and fibronectin all form nanofibrous structures in native ECM that play a key role in regulating cell behavior (Stevens and George, 2005), particularly in terms of promoting cell adhesion and spreading. The internal porous structure of a hydrogel is also critical to tune the transport of nutrients, gases, and wastes within the tissue and providing sufficient physical space for cells to communicate and ultimately form a tissue (Atala, 2014; de France et al., 2021). To obtain such structured hydrogels, traditional methods such as emulsion templating (Zhang and Cooper, 2005; Partap et al., 2006), gas foaming (Harris et al., 1998), salt leaching (Lee et al., 2005), and cryogelation (Vrana et al., 2009) have been used to prepare scaffolds with different pore size distributions (Figure 3). However, the required use of at least one of solvents, additives, or external energy in each of these strategies significantly impedes the potential for the direct encapsulation of cells and *in vivo* cell delivery; control over the pore size and shape is also very challenging with these methods, creating a situation in which cells in different parts of the scaffold receive different cues from their microenvironment. Photopatterning (Liu and Bhatia, 2002; Chan et al., 2010) and micro-molding (Hammer et al., 2014; Liu et al., 2014). can address these challenges but are low-throughput techniques that can significantly limit the size and/or the number of scaffolds that can be practically fabricated. In the following section, we will emphasize on three emerging fabrication techniques for preparing hydrogel-based tissue scaffolds with well-defined pore structures that are both scalable in the context of the materials and crosslinking strategies previously discussed as well as cell-friendly to enable simultaneous structure formation and cell loading (as is essential for effective translation): 3D bioprinting, cell electrospinning and *in situ* tissue engineering.

### 3.1 3D Bioprinting

**FIGURE 4:**
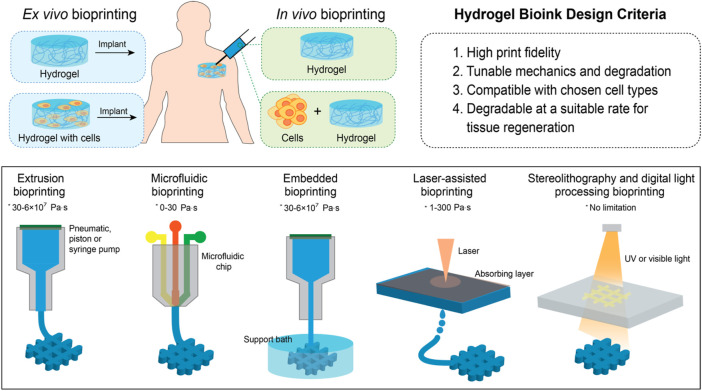
Schematic of *ex vivo* and *in vivo* bioprinting techniques (* represents the viscosity range of bioinks useful for each bioprinting technique).

Three-dimensional (3D) bioprinting is an additive manufacturing process that creates highly-complex tissue constructs using the layer-by-layer deposition of biomaterials onto a computer-controlled build platform (Pedde et al., 2017). 3D bioprinting allows for the fabrication of complex hydrogel geometries that can be loaded with active components such as drugs, growth factors, or viable cells directly during the fabrication process (Figure 4). The automated nature of 3D bioprinting enables the fabrication of both reproducible and scalable structures, both of which are key challenges with conventional structuring strategies. Many variations of 3D bioprinting including extrusion bioprinting (Ramesh et al., 2021), microfluidic bioprinting (Zhang et al., 2017), inkjet bioprinting (Li et al., 2020), digital light processing (DLP) bioprinting (Wang et al., 2021), laser-assisted bioprinting (Guillotin et al., 2010), stereolithography (SLA) bioprinting (Grigoryan et al., 2021), and embedded bioprinting (Shiwarski et al., 2021) have been developed, each of which has its own optimal set of bioink materials, advantages and disadvantages, and most promising clinical applications. For hydrogels, inkjet bioprinting is typically avoided given the high viscosity of many hydrogel-based bioinks; however, hydrogels with well-defined shapes and internal morphologies have been widely printed using each of the other techniques. Note that, in the following sections, the term “biomaterial ink” is used to refer to a biomaterial printed alone and then subsequently seeded with cells while the term “bioink” is used to refer to cases in which a biomaterial is (or could be) co-printed with one or more cell(s).

#### 3.1.1 Extrusion Bioprinting

In extrusion bioprinting, a hydrogel or hydrogel precursor bioink is extruded out of a narrow print-tip such as a needle or conical nozzle that is moving through space to build a 3D structure layer by layer. The flow of the hydrogel can be driven by pneumatics (Duarte Campos et al., 2019; Ouyang et al., 2020) or mechanical flow driven by a syringe pump or screw mechanism (Liu et al., 2017). The tunable rheological properties (Camci-Unal et al., 2013; Antich et al., 2020) and controllable gelation rates (Wang et al., 2018; Xu et al., 2018) of hydrogels make them well-suited for extrusion-based bioprinting, particularly for shear-thinning hydrogels that can maintain their shape upon extrusion; however, low-viscosity hydrogels with controllable gelation rates can also be used for extrusion bioprinting if gelation of the hydrogel can be induced during or immediately after extrusion such that the print will maintain its geometry (Ramesh et al., 2021).

The most prevalent bioink used in extrusion bioprinting is the alginate/calcium chloride system, performing the crosslinking either via a coaxial nozzle in which alginate and calcium chloride are delivered in separate streams (Zhang et al., 2013; Mirani et al., 2017) or by printing the alginate into a calcium chloride bath (Tabriz et al., 2015). However, most of the useful hydrogel materials outlined earlier do not inherently have rapid gelation mechanisms like calcium/alginate, driving the development of more advanced approaches such as using hybrid hydrogels consisting of homogeneous mixtures of multiple hydrogels or using coaxial extruders to create fibers with a distinct core and sheath from separate materials (Tamayol et al., 2015); in many cases, alginate-calcium is still used as the primary gelling component to entrap/encapsulate other functional components (Onoe et al., 2013). For example, Antich et al. bioprinted chondrocyte-laden hyaluronic acid (HA) *in vitro* by mixing alginate with HA to allow for rapid ionic crosslinking immediately after printing while maintaining a HA-rich hydrogel phase (Antich et al., 2020) while Liu et al. (2018) developed a coaxial bioprinting strategy with alginate in the sheath and GelMA, cells, and calcium chloride in the core channels (Liu et al., 2018); in the latter case, the rapidly crosslinked and comparatively strong alginate sheath allowed for the use of very low (1% w/v) GelMA concentrations in the core which are very desirable for cell proliferation but too mechanically fragile and slow to crosslink to print directly (Liu et al., 2018). A major challenge with extrusion bioprinting however is the shear stress in the nozzle which can be detrimental to cell viability and function. Higher viscosity hydrogels, that are often used for extrusion bioprinting due to their better printability, typically require higher pressures to extrude, leading to even higher shear stresses. In addition, the resolution of the extruded features in hydrogel-based bioinks is typically low (i.e., on the several tens to hundreds of micron length scale), much higher than the nanoscale features that predominantly regulate cell responses in the native ECM. As such, the development of new extrusion printing approaches/geometries to reduce the minimum printed feature size may be highly impactful. One potential strategy to address this drawback without requiring the development of entirely new printing strategies may be the use of jammed microgel-based bioinks, in which the size and softness of the hydrogel microparticles printed creates a specific self assembly/packing pattern that results in a specific pore size between the jammed microparticles on a length relevant to the dimensions of native ECM features. For example, Xin et al. demonstrated the thiol-ene photopolymerization of packed electrosprayed PEG-microgels (∼200 µ μ m in diameter) using a low concentration PEG-dithiol linker to create a permissive 3D environment that enabled spreading of human mesenchymal stem cells (Xin et al., 2018) while Hou et al. fabricated macroporous hydrogels by covalently crosslinking gelatin microgels (∼250 µ μ m in diameter) with microbial transglutaminase that allowed for enhanced proliferation of human dermal fibroblasts over 2 weeks and enhanced migration of cells into the scaffold both *in vitro* and *ex vivo* (Hou et al., 2018). Click chemistry approaches have also been demonstrated to link the microgels together in the presence of cells without the need for post-loading of cells. For example, Caldwell et al. reported the *in situ* gelation of both ∼10 µ μ m and ∼100 µ μ m microgels functionalized with azide and dibenzocyclooctyne (DBCO) groups in the presence of human mesenchymal stem cells, with the 100 µ μ m microgel networks creating a network of fewer but larger pores that promoted significantly more cell spreading (Caldwell et al., 2017). Deveoping methods to improve the mechanics of such jammed microgel bioinks such that the networks are stiffer and/or more stable over longer-term implantation would further expand the scope of using such bioinks in the context of functional extrusion bioprinting.

#### 3.1.2 Microfluidic Bioprinting

**FIGURE 5:**
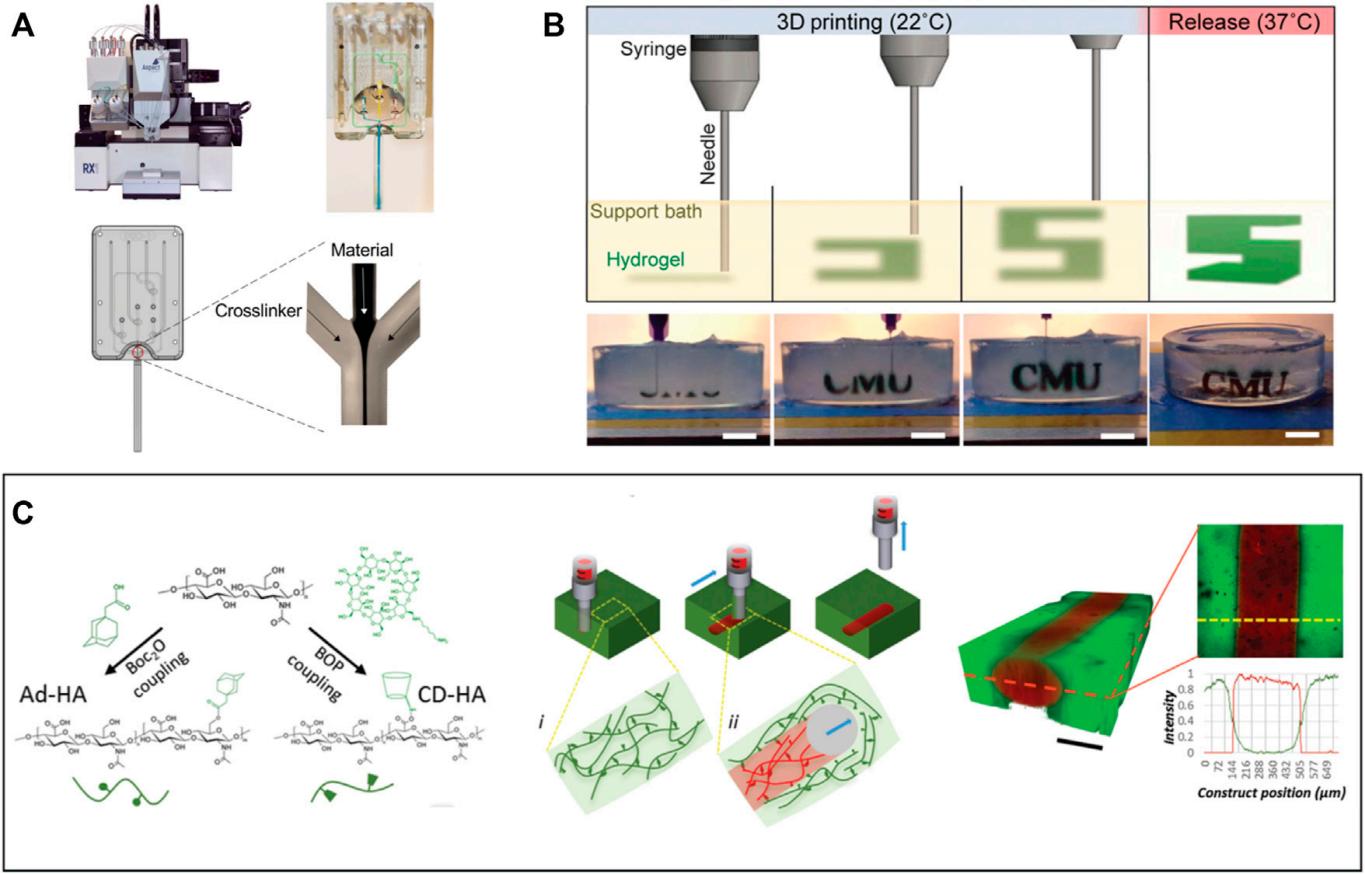
Examples of emerging 3D printing approaches: **(A)** 3D bioprinting system with a microfluidic printhead that can load multiple biomaterials in different channels (Reproduced with permission from Dickman et al., 2020). **(B)** Schematic and images of FRESH printed alginate gels embedded in gelatin slurry bath. Scale bar = 1 cm (Reproduced with permission from Hinton et al., 2015). **(C)** Extruded FRESH printing of HA hydrogel into self-healing support hydrogel bath. Scale bar = 200 μm (Reproduced with permission from Highley et al., 2015).

Microfluidic chips, typically fabricated with polydimethylsiloxane (PDMS) (Colosi et al., 2016; Feng et al., 2019; Dickman et al., 2020) or micro-milled surfaces (Costantini et al., 2017), enable hydrogels to be mixed, crosslinked, or otherwise manipulated upstream from the print tip. The channel pattern on microfluidic chips is highly customizable, making microfluidic bioprinting a good strategy for addressing many difficult biofabrication problems. In addition, microfluidic bioprinting allows for seamless switching between different materials during fabrication using either programmable syringe pumps connected to the hydrogel inputs (Feng et al., 2019), or valves actuated by pneumatics (Colosi et al., 2016; Addario et al., 2020). Microfluidic bioprinting solutions that incorporate more complicated microfluidic geometries or channel junctions are commercially available from Fluicell and Aspect Biosystems. Fluicell’s microfluidic bioprinter and handheld biopen can fabricate 2D or 3D cell-laden structures with control over single-cell deposition (Jeffries et al., 2020), particularly useful for conducting pre-clinical testing of valuable drugs on cells in 3D environments. Aspect Biosystems’ microfluidic bioprinter allows for rapid switching between multiple cell-laden hydrogels and contains a microfluidic junction enabling mixing of crosslinkers with precursor polymers (e.g., alginate and calcium) shortly before extrusion (Figure 5A) (Dickman et al., 2020). By modifying the crosslinking and precursor polymer solutions, this microfluidic chip can also bioprint chemically crosslinkable thrombin (Abelseth et al., 2019; Lee et al., 2019) or photo-crosslinkable bioinks (Mirani et al., 2021). In general, microfluidics bioprinting benefits from the wide array of operations that can be performed on a microfluidic chip prior to bioprinting, allowing for highly controlled and dynamic mixing of multiple components over time. However, the fabrication, testing, and optimization of microfluidic chips is a lengthy process that requires expensive and specialized equipment and the microfluidic print heads can be considerably more expensive than other print heads, particularly in cases in which the print head itself is designed as a consumable unit.

#### 3.1.3 DLP and SLA Bioprinting

In digital light processing (DLP) bioprinting, a photosensitive hydrogel is crosslinked by patterns of light in a layer-by-layer approach. Typically, the build platform is lowered into a hydrogel precursor solution placed on top of a transparent substrate and a light source. A thin layer of the hydrogel is exposed to a light pattern, the build platform is raised a small distance, and the process is repeated until the print is complete. The light pattern for each layer can be produced by a photomask (Shen et al., 2020), projector (Grigoryan et al., 2019; Mahdavi et al., 2020), or digital micromirror device (DMD) (Rujing and Larsen, 2017; Wang et al., 2018). A major benefit of this technique is that it harnesses the high spatial resolution of projectors or DMDs to produce constructs with spatial resolutions of <100 mm in the horizontal plane (Wang et al., 2018; Grigoryan et al., 2019; Magalhaes et al., 2020), a notable improvement compared to most extrusion bioprinting. Additionally, DLP bioprinting does not have issues with layer adhesion, is well suited to produce constructs with perfusable channels (Grigoryan et al., 2019), and can produce large constructs quickly as entire layers crosslink simultaneously. Stereolithography (SLA) bioprinting uses a similar concept except the light source is a laser which photo-crosslinks a single point at each moment in time instead of a single layer, using mirrors angled to reflect the laser across the corresponding areas of the build platform. While such an approach can create even more complex geometries with superior resolutions, it is correspondingly much slower given that only a single laser cross-section can be crosslinked at any given time, posing challenges with scale-up manufacturing of particularly larger tissue scaffolds. Aside from speed, the main constraint of DLP and SLA bioprinting is the limitation of hydrogel types that can be used. Although any polymer functionalized with a photopolymerizable functional group could in principle be bioprinted using DLP/SLA bioprinters, commonly used hydrogels for DLP or SLA bioprinting include gelatin methacryloyl (GelMA), poly (ethylene glycol) diacrylate (PEGDA) and hyaluronic acid methacrylate (HAMA) (Wang M. et al., 2021; Grigoryan et al., 2021). PEGDA currently provides the best spatial resolution of all biocompatible photo-crosslinkable hydrogels, however, it does not promote cell attachment and alone is not suitable as a matrix for tissue engineering applications. GelMA offers slightly lower resolution, but its high capacity for cell adhesion has resulted in successful DLP and SLA printing of corneal stroma (Mahdavi et al., 2020), cartilage (Lam et al., 2019), and vasculature (Tomov et al., 2021).

#### 3.1.4 Embedded Bioprinting

Soft hydrogels provide a favorable environment for cells to grow, proliferate and differentiate (He et al., 2016; Ramiah et al., 2020); however, the weak mechanics of such hydrogels results in challenges maintaining print fidelity/avoiding structure collapse when printed on a platform directly. Embedded bioprinting addresses this challenge via the use of gel-based support baths to support extrusion printing of soft hydrogels into higher-resolution 3D constructs (McCormack et al., 2020). Highly shear-thinning but viscous materials such as Carbopol (Bhattacharjee et al., 2015), gellan (Compaan et al., 2019), agarose (Mirdamadi et al., 2019), and gelatin microparticles (Hinton et al., 2015) have been most commonly reported as the support bath material. The freeform reversible embedding of suspended hydrogels (FRESH) printing process that uses gelatin microparticles as the support bath, pioneered in 2015 by the Feinberg, Angelini and Burdick research groups (Figures 5B,C), has been particularly successful in enabling the printing of inks with a broad range of rheological properties (Bhattacharjee et al., 2015; Highley et al., 2015; Hinton et al., 2015; Shiwarski et al., 2021). In this process, the bioink is printed directly into the gelatin support bath, crosslinked, and subsequently placed in physiological temperature to liquify the gelatin support bath to release the printed construct. The most common crosslinking strategies include ionic crosslinking [e.g., alginate/calcium (Compaan et al., 2019; Lindsay et al., 2019)], photocrosslinking [e.g., methacrylated or acrylated pre-polymer solutions (Ouyang et al., 2017)], and post-modification by changing the solution conditions [e.g., a change in pH for collagen gelation (Isaacson et al., 2018; Noor et al., 2019)]. Embedded bioprinting overcomes challenges with printing soft hydrogels and incorporating viable cells using a low-shear printing process but remains amenable to a relatively low diversity of bioinks, the potential for highly inhomogeneous crosslinking (particularly when the crosslinker is placed in the support bath), and scale-up to allow for larger printed constructs, particularly given the significant costs of some of the support bath materials.

### 3.2 Electrospinning

In electrospinning, a high voltage difference is applied between a needle extruding a precursor polymer solution and a conductive collector. When the electrostatic repulsion forces of the charged polymer solution overcome the surface tension of the polymer solution, a fiber is ejected (Reneker and Yarin, 2008) that can be collected on a grounded conductive collector, which may include rotating drums, flat collectors, or parallel collectors (Li et al., 2003; Katta et al., 2004; Xu et al., 2016). The versatility and relatively low experimental complexity of the electrospinning setup makes it an accessible and feasible method of forming polymer nanofibers that have been widely used in a variety of tissue engineering applications (Greiner and Wendorff, 2007; Sill and von Recum, 2008). The recent development of portable handheld electrospinning devices (in contrast to conventional devices that require a large power supply) has further expanded the potential of this technique (Brako et al., 2018), enabling the direct *in situ* application of polymeric nanofibers (to-date including PCL, polystyrene (PS), poly (lactic acid) (PLA) and poly (vinylidene fluoride) (PVDF)) in the clinic for wound healing and other applications (Xu et al., 2015).

Electrospinning requires careful tuning of several parameters including polymer concentration, solvent, relative humidity, high voltage, collector, working distance, solution viscosity, and flow rate (Xue et al., 2019). When considering the electrospinning of hydrogels, crosslinking and gelation kinetics must also be considered, particularly relative to their effects on the polymer solution viscosity throughout the electrospinning process (Xu et al., 2016); in particular, it is imperative to maintain a flowable solution at the needle outlet but produce a stable crosslinked fiber (or a sufficiently viscous fiber that buys time for covalent or physical crosslinking to occur) at the collector. Some methods of hydrogel electrospinning instead elect to crosslink as a post-processing step of the nanofiber scaffold to avoid changes in viscosity to the electrospinning solution during the fabrication process itself; however, this choice adds an additional step to the process and may result in deformation of the original electrospun structure on the collector prior to the completion of the crosslinking process (Deng et al., 2018).

Electrospun hydrogels based on both natural polymers such as collagen, gelatin, dextran, alginate, HA, or chitosan as well as synthetic polymers such as POEGMA (Xu et al., 2016) have been reported, with other synthetic polymers such as poly (ethylene oxide) (PEO), poly (vinyl pyrrolidone) (PVP), or poly (vinyl acetate) (PVA) also in some cases included to promote chain entanglement in the precursor polymer solution and thus nanofiber formation instead of particle sprays. Stiffer degradable polymers such as poly (ε-caprolactone) (PCL) may also be added to increase the mechanical strength of the porous hydrogel scaffold, a key challenge with electrospinning (Koosha and Mirzadeh, 2015; Xu et al., 2016; Majidi et al., 2018; Wakuda et al., 2018; Askarzadeh et al., 2020). Table 1 summarizes different materials and methods used for electrospinning hydrogels. Common crosslinking methods include chemical crosslinking through saturation of the scaffold with the chemical crosslinking agent, photo-crosslinking with UV-light irradiation, and physical crosslinking such as the ionic crosslinking of calcium-alginate hydrogels (Gombotz, 1998; Hennink and van Nostrum, 2012), methods that will be further described in the following sections.

**TABLE 1:** Materials and methods used for electrospinning hydrogels for tissue engineering.

	Materials	Solvent(s)	Crosslinking mechanism(s)	Voltage (kV)	Cells incorporated	Nanofiber size (nm)	Key biological results	Specific application(s)	References
Natural Polymers	Collagen	Hexafluoro-2-propanol	Chemical (EDC-NHS, genipin, transglutaminase, UV photo-crosslinking)	11	Human osteosarcoma MG-63 cells	106 ± 22	• Faster cell growth on EDC/NHS crosslinked scaffolds compared to TG or GP-crosslinked scaffolds over up to 21 days	Bone tissue engineering	Torres-Giner et al. (2009)
	Collagen	Hexafluoro-2-propanol and acetic acid	Chemical (EDC-NHS, glutaraldehyde, genipin)	20	Mc3T3-E1 cells	300–650	• Best cell proliferation observed using EDC-NHS as the crosslinker	Extracellular matrix model	Luo et al. (2018)
	Collagen and chitosan	Acetic acid and ethanol	Chemical (EDC)	16	HUVECs and NIH 3T3 fibroblast cells	168 ± 58	• Facilitated improved cell viability when compared to the tissue culture dish control	Wound healing	Deng et al. (2018)
	Gelatin	Acetic acid, ethyl acetate and water	Chemical (glutaraldehyde, genipin, glyceraldehyde, reactive oxygen species)	12	MG63 osteoblast cells	∼300	• Glyceraldehyde-crosslinked nanofibers maintained highest cell viability and growth	Tissue engineering	Sisson et al. (2009)
	Gelatin	Acetic acid and water	Chemical (EDC, genipin, GTA vapour)	15	HeLa epithelial cells	268 ± 18	• EDC/NHS crosslinking resulted in the longest stability in a physiological-like environment	Tissue engineering	Campiglio et al. (2020)
	Methacrylated dextran	Sodium bicarbonate and HEPES	Chemical (UV photocrosslinking)	7.5	NIH 3T3 fibroblasts and human mesenchyme stem cells	<500	• Fiber scaffold stiffness did not affect cell viability, but remodeling of the scaffold occurred to a much higher degree in soft scaffolds	Extracellular matrix model	Baker et al. (2015)
	GelMA	Hexafluoro-2-propanol	Chemical (UV photocrosslinking)	15	Bone mesenchymal stem cells and hippocampal neuronal cells	∼1000	• Decreased glial scar tissue formation, increased vascularization, and increased neuronal development compared to electrospun gelatin fibers crosslinked with glutaraldehyde	Spinal cord regeneration	Chen et al. (2019)
Natural and Synthetic Polymers	Alginate and PEO	Triple-distilled water	Physical (ionic crosslinking via CaCl_2_)	10.5	C2C12 myoblast cells	250–400	• >90% cell viability over 7 days	Skeletal muscle tissue regeneration	Yeo and Kim (2018)
							• Cells grow along the direction of the aligned fibers		
	Alginate, PEO, GelF-MA with Pluronic^®^ F127	Deionized water	Dual (ionic crosslinking with CaCl_2_ + UV photocrosslinking)	7	Mesenchymal stem cells	183 ± 36	• < 10% cytotoxicity and an 8-fold increase in cell proliferation observed over 2 weeks	Stem cell therapy and tissue regeneration	Majidi et al. (2018)
							• Signs of maturation of the human iPSC-derived ventricular cardiomyocytes observed		
	Gelatin-hydroxyphenyl-propanoic acid (Gel–HPA)	Hexafluoro-2-propanol and water	Enzymatic (oxidation of the HPA moieties with the addition of horseradish peroxidase and H_2_O_2_.)	18	HUVECs	∼ 400–2,000	• Full scaffold degradation observed within 4 weeks of *in vivo* implantation with good cell penetration	Soft tissue engineering	Nie et al. (2020)
	Thiolated hyaluronic acid (HA) and PEO	DMEM cell medium	Chemical (disulfide formation + thiol-Michael addition following the post-fabrication addition of PEGDA)	18	NIH 3T3 fibroblast cells	50–300	• Cells can infiltrate the scaffold up to 32 µm below the surface and showed an extended dendritic network morphology compared to 2D controls	Cell encapsulation and tissue regeneration	Ji et al. (2006)
	Alginate and PEO	Triple-distilled water	Physical (ionic crosslinking using CaCl_2_)	10.5	HUVEC, C2C12, or H9c2 smooth muscle cells	328 ± 50–488 ± 67	• 90% cell viability maintained	Muscle tissue regeneration	Yeo and Kim (2020)
							• Myogenic gene expression markers identified		
							• 2,154% increase in cell proliferation with HUVECs and seeded C2C12 cells		
	Fibrinogen and PEO	Deionized water	Chemical (thrombin-induced crosslinking)	4.5	C2C12 myoblast cells	80,000–90,000	• Higher viability achieved by electrospinning aggregates and decreasing voltage	Muscle tissue regeneration	Guo et al. (2019)
							• Induced myogenesis of C2C12 aggregates growing along microfiber bundle		
	Collagen and PVP	Hexafluoro-2-propanol	Physical (pH-induced)	3.6	HUVECs	461 ± 129	• Altered crosslinking methods maintained the triple helical structure of collagen through the electrospinning process	Tissue engineering	Wakuda et al. (2018)
							• HUVECs cultured on scaffolds along the fiber direction		
	Chitosan and PVA	Acetic acid	Physical (temperature-induced)	20	L-929 fibroblast cells	172 ± 60–257 ± 63	• Attachment and proliferation of fibroblast cells over 5 days	Wound healing and tissue engineering	Koosha and Mirzadeh (2015)
	Collagen and PVA	Acetic acid and water	Dual (phosphoric acid + glutaraldehyde)	12–15	Human keratocytes (HKs) and human corneal epithelial cells (HCECs)	163–211	• HKs align to the fiber orientation	Cornea tissue engineering	Wu et al. (2018)
							• Good cell adhesion and proliferation over 4 weeks		
	Chitosan and PVA	Acetic acid and water	Chemical (glyoxal)	15	Human normal fibroblast cells	227 ± 63	• 3.5× increased strength with 5% halloysite nanotubes (HNTs) incorporated into the fibers	Skin tissue regeneration	Koosha et al. (2019)
							• HNTs-reinforced fibers exhibited better cell attachment on surface of nanofibers		
	Hyaluronic acid, PVA, l-arginine and cellulose nanocrystals (CNCs)	Water	Physical (citric acid)	28–30	Human normal lung fibroblast WI38 and skin melanocyte HFB-4 cells	122–222	• Increased fiber mechanical strength due to CNC addition	Wound healing and tissue engineering	Hussein et al. (2020)
							• Increased ECM collagen synthesis, angiogenesis, and epithelialization		
Synthetic Polymers	Poly (oligoethene glycol methacrylate) (POEGMA) and PEO	DMEM cell medium and PBS	Chemical (*in situ* hydrazone crosslinking)	10	NIH 3T3 fibroblast and C2C12 myoblast cells	∼400	• Cells can be encapsulated directly within POEGMA hydrogel nanofibers during electrospinning without any pre- or post-treatment	Cell encapsulation and tissue engineering	Xu et al. (2018)
							• High cell viabilities after cell electrospinning and 3–4× cell proliferation over 18 days		

#### 3.2.1 Photocrosslinked Hydrogel Fibers

Methacrylated natural polymers such as alginate, gelatin and dextran have been electrospun to form photocrosslinked hydrogel scaffolds. Photocrosslinking can be used as the only crosslinking strategy or (depending on its speed) a secondary crosslinking step toward producing a multi-crosslinked hydrogel network (Chen et al., 2018). For example, alginate, GelMA, PEO, and the photoinitiator Irgacure 2959 were electrospun, placed in a CaCl_2_ bath (enabling rapid primary alginate-calcium ionic crosslinking) and subsequently exposed to 10 min of UV irradiation (enabling secondary GelMA photo-crosslinking). High cell viability (>90%) could be maintained coupled with an 8-fold increase of cell number in human iPSC-derived ventricular cardiomyocytes in 3D culture over 2 weeks of observation (Majidi et al., 2018).

For soft tissues, photocrosslinking can be used directly to form the hydrogels. For example, GelMA dissolved in the solvent hexafluoroisopropanol (HFIP) was reported to fabricate aligned electrospun nanofibers that were subsequently submerged in anhydrous alcohol containing Irgacure 2959 and crosslinked under UV light for 60 min to form hydrogel nanofibers (Chen et al., 2019). *In vivo* work showed that implantation of the GelMA scaffolds in rats enabled decreased glial scar tissue formation, increased vascularization, and increased neuronal development compared to electrospun gelatin fibers crosslinked with glutaraldehyde. The degree of crosslinking can also be tuned by adjusting the UV-light exposure time post-fabrication. Baker et al. (2015) similarly showed that DexMA could be electrospun to form a hydrogel scaffold with a range of scaffold moduli based on the UV exposure time. While the fiber scaffold stiffness did not affect cell viability, remodeling of the scaffold occurred to a much higher degree in the soft scaffolds (Baker et al., 2015). However, it should be noted that there is considerable debate over the degree to which UV irradiation may impact encapsulated cells (both in the short term and the long term). While the wavelength and the total dose (intensity + time) of the irradiation certainly does influence the degree to which UV irradiation may impact encapsulated cells, access to alternative crosslinking strategies (particularly for cells that have less robust viability *in vitro*) is recommended.

#### 3.2.2 Chemically Crosslinked Hydrogel Fibers

Electrospun hydrogels can also be formed by chemically crosslinking the polymer components via covalent bond forming chemistries. Common chemical crosslinking agents include N-(3-dimethylaminopropyl)-N′-ethylcarbodiimide hydrochloride (EDC, for crosslinking proteins), genipin (for crosslinking aminated polymers), glutaraldehyde (GTA, for crosslinking hydrazide and aminated polymers), and glyoxal (for crosslinking hydroxylated polymers) (Luo et al., 2018; Koosha et al., 2019). Such crosslinking agents are typically added either in liquid or vapor form following electrospinning to crosslink the hydrogel fibers (Sisson et al., 2009), with the typically rapid hydration rate of the electrospun hydrogel when exposed to a moist or saturated environment potentially deforming the fibers (via swelling) on the same time scale as crosslinking. In addition, the cytotoxicity of many of these chemical crosslinking agents is a significant concern, with many studies having been published seeking to define the optimum type and concentration of crosslinker for maintaining high cell viability (Hennink and van Nostrum, 2012; Luo et al., 2018; Campiglio et al., 2020). For example, Deng et al. (2018) examined the use of low concentrations of EDC co-electrospun recombinant human collagen (RHC), chitosan, and PEO in an acetic acid and ethanol solution to aid in solvent evaporation and dry fiber formation. The slow gelation that occurs at the low EDC concentration used results in the fabrication of hydrogel nanofibers with diameters of 168 ± 58 nm that facilitated improved seeded NIH 3T3 and human umbilical vein endothelial cell (HUVEC) cell viability when compared to the tissue culture dish control (Deng et al., 2018); however, the fabrication of thicker scaffolds (requiring longer electrospinning times) may be challenging when the crosslinker is added directly to the electrospinning solution. As another example, genipin, EDC-NHS, and glutaraldehyde were compared to assess which crosslinker best maintained the triple helical structure of electrospun collagen fibers (Luo et al., 2018), with the best cell proliferation observed using EDC-NHS as the crosslinker (although all three scaffolds exhibited improved cell proliferation relative to the control) (Luo et al., 2018). Similarly, Torres-Giner et al. electrospun collagen dissolved in HFP, post-crosslinked the fibers with EDC-NHS, and then seeded with the scaffold with MG-63 cells, enabling faster cell proliferation and far more cell growth over 21 days compared to a genipin-crosslinked scaffold (Torres-Giner et al., 2009). Genipin, GTA vapour, and glyceraldehyde have also been specifically compared by Sisson et al. as crosslinking agents for electrospun gelatin fibers, with glyceraldehyde-crosslinked nanofibers found to maintain the highest cell viability and growth (Sisson et al., 2009). However, any small molecule crosslinker that reacts non-bioorthogonally with proteins in or secreted from cells does offer some risk in terms of promoting undesired cell or (following implantation if the crosslinker is not thoroughly removed) tissue toxicity and should be used only judiciously.

Chemical crosslinking with GTA has also been explored in comparison to the physical crosslinking method of dehydrothermal treatment (DHT) for fabricating an electrospun collagen scaffold (Chen et al., 2021). The triple helix in the collagen fibers was better maintained when using chemical crosslinking methods, with the use of GTA avoiding the need for heat application as is required with the DHT method (Chen et al., 2021). Ammonia treatment of the collagen electrospun scaffold after fabrication to neutralize any remaining acetic acid further improved maintenance of the triple helix collagen structure in the fibers (Chen et al., 2021). Using a volatile crosslinker can also facilitate both penetration throughout the scaffold and post-purification of the scaffold to ensure the removal of unreacted crosslinker; for example, a PVA/collagen blend electrospun in HFIP and crosslinked under phosphoric acid vapor followed by GTA vapor promoting suitable seeded cell viabilities and transparency for corneal tissue engineering applications (Wu et al., 2018).

Covalent chemical crosslinking can also enable more efficient entrapment of additives into the electrospun hydrogels without significantly compromising the crosslinking and mechanical strength of the scaffold. For example, Hussein et al. (2020) electrospun HA, PVA, cellulose nanocrystals (CNCs), and l-arginine to form hydrogel fibers with the PVA component chemically crosslinked through the addition of anhydrous citric acid prior to electrospinning. CNC addition serves to increase the mechanical strength of the fibers while l-arginine promotes ECM collagen synthesis, angiogenesis, and epithelialization, promoting improved cell viabilities when cells were seeded on the scaffold. The scaffold also had anti-microbial properties related to l-arginine release and faster wound closure times compared to controls. However, the inclusion of multiple components in the electrospinning solution that may cross-interact in multiple ways can pose challenges with controlling the precursor solution viscosity and thus the uniformity of the electrospun hydrogel.

Enzymes can also be used as chemical crosslinking agents to minimize cytotoxicity. For example, a gelatin-hydroxyphenylpropanoic acid (Gel–HPA) scaffold electrospun in hexafluoroisopropanol (HFIP) was crosslinked via enzymatic oxidation of the HPA moieties with the addition of horseradish peroxidase and H_2_O_2_ (Nie et al., 2020). Enzymatic crosslinking scaffold preparations reported full scaffold degradation within 4 weeks of *in vivo* implantation with good cell penetration (Nie et al., 2020); however, the specific substrates required plus the cost of most enzymatic crosslinkers may limit the broad utility of this approach in the context of a larger manufacturing platform.

#### 3.2.3 Solvent-Free Electrospun Hydrogels

While the use of organic solvents can be a useful tool in the formation of electrospun nanofibers due to the ease of solvent evaporation between the nozzle and the collector, emerging studies have focused on electrospinning polymer solutions in water to remove the need for solvent removal before cell seeding and do not risk exposure of the seeded cells to cytotoxic crosslinking chemicals or photoinitiators. Altered experimental setups and polymer chemistries have both been explored as methods to reduce the need for electrospinning with organic solvents. First, the use of sacrificial sheaths can be used. Wakuda et al. (2018) reported a core-shell electrospinning approach in which the outer layer contained PVP while the inner layer consisted of collagen pre-crosslinked in a basic solution. Post-fabrication, the PVP layer was dissolved to leave the insoluble collagen, avoiding challenges associated with maintaining the anisotropic triple helix structure in organic solvents (Torres-Giner et al., 2009; Luo et al., 2018). HUVECs cultured on the surface of the formed collagen fibers followed the orientation of the fibers, unlike HUVECs seeded on collagen fibers electrospun in HFIP directly (Wakuda et al., 2018). Second, latent click or click-like chemistries can be used to lock the printed nanofiber in place after electrospinning. For example, Ji et al. (2006) electrospun thiolated HA with PEO as an electrospinning aid in DMEM, enabling chemical crosslinking via both disulfide formation and thiol-Michael addition following the post-fabrication addition of poly (ethylene glycol)-diacrylate (PEGDA). 3T3 fibroblasts seeded on the scaffolds were found to infiltrate the scaffold up to 32 mm below the scaffold surface and showed better morphology compared to 2D cell culture controls (Ji et al., 2006). However, this approach still required a post-treatment of a pre-electrospun nanofiber network. Third, reactive electrospinning techniques have been developed in which *in situ-*gelling polymer pairs are co-delivered through a double-barrel syringe directly into the electrospinning process. For example, Xu et al. (2016) electrospun aldehyde and hydrazide-functionalized POEGMA polymers from a double-barrel syringe tuned to have gelation times that ensured free flow of the polymers at the needle tip but rapid gelation upon electrospinning, leveraging the acceleration of gelation rate as water evaporates and the polymers concentrate in the emitted jet; as such, the nanofibers are sufficiently gelled upon hitting the collector that their structure can be maintained without the need for any post-processing/post-crosslinking step (Xu et al., 2016). Of note, conducting the process in a biosafety cabinet can also directly produce sterile scaffolds without any additional sterilization requirement. Both protein-repellent and thermo-responsive electrospun POEGMA scaffolds were prepared by varying the polymer component ratios, with the latter found to expand and contract reversibly to facilitate cell adhesion (at physiological temperature) but rapid cell delamination within 2 min upon swelling of the scaffold at 4°C that could serve as a replacement for typical trypsin-based cell delamination methods (Xu et al., 2021).

#### 3.2.4 Cell Electrospinning

**FIGURE 6:**
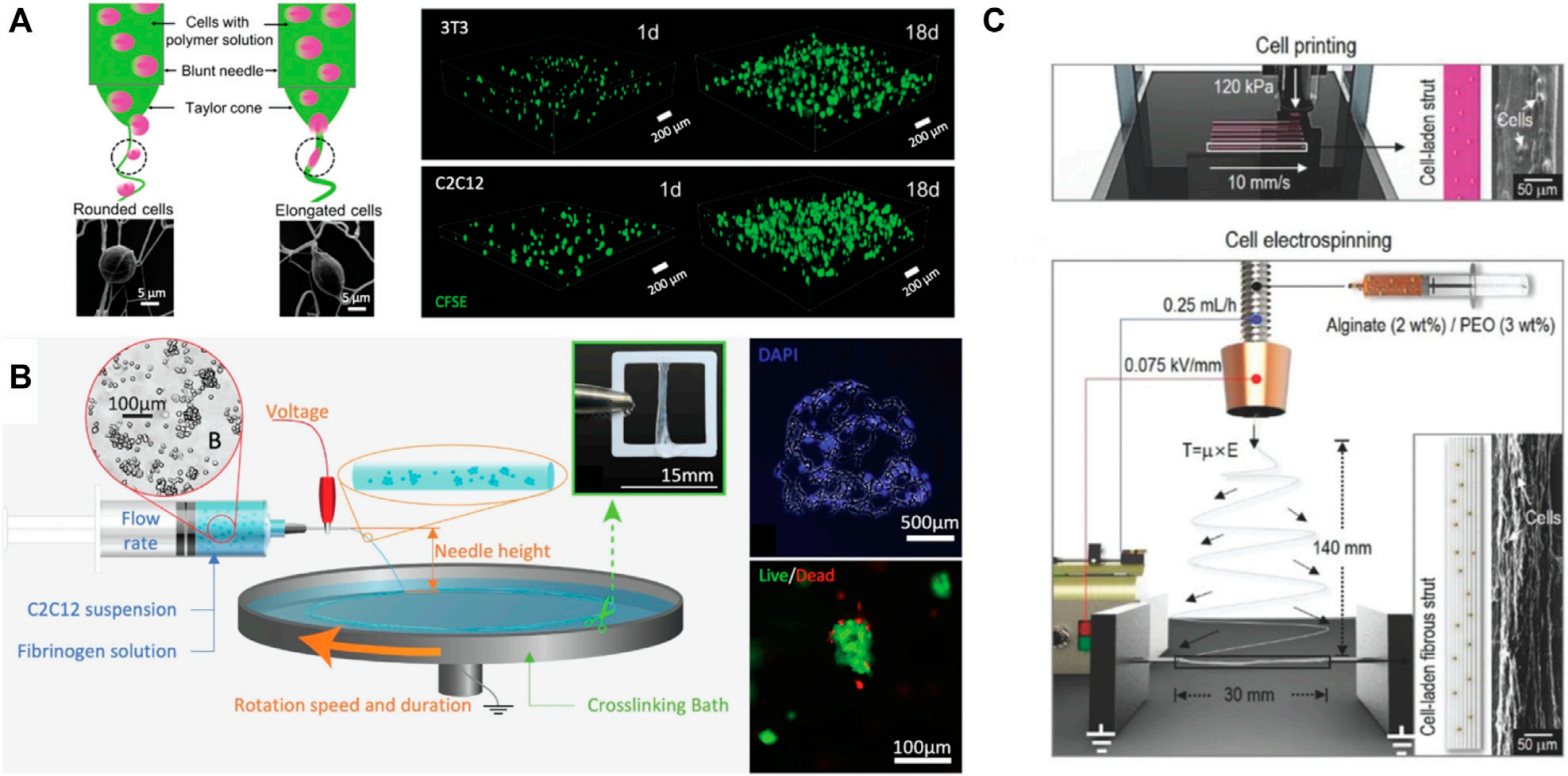
Strategies for cell electrospinning: **(A)** reactive electrospinning of 3T3 mouse fibroblasts and C2C12 mouse myoblasts in POEGMA hydrogel nanofibers; **(B)** cell-laden electrospinning of C2C12 myoblasts in fibrin scaffolds; **(C)** combining cell printing and cell electrospinning to encapsulate C2C12 myoblasts in alginate scaffolds. Reproduced with permission (Xu et al., 2018; Yeo and Kim, 2018; Guo et al., 2019).

All examples described to this point required separate fabrication and cell loading steps, with the use of solvents, the drying of the scaffold, and/or the challenges with conducting electrospinning in a sterile environment all limiting cell survival in addition to the shear and electric field stress placed on cells during the electrospinning process. Relatively few examples exist of combining these steps together, which is highly beneficial to avoid the need to seed cells onto the scaffold post-fabrication and introduce more flexibility into the electrospinning process (akin to 3D printing) in terms of structuring different cell types within a single fabricated scaffold. The reactive electrospinning technique (Figure 6A) offers particular advantages in this regard given that no additional post-processing is required, enabling returning cells to an incubator in sterile conditions much faster than with other techniques, and the entire process is designed to run in water. The cell-friendly and high water-binding nature of the POEGMA electrospun fibers enabled high survivals of >80% of both NIH 3T3 fibroblasts and C2C12 myoblasts, survivals maintained even following cryoprotectant-free storage of the visibly “dry” scaffolds in liquid nitrogen over a 3 week period; in addition, 3-4x increases in cell number were observed over the 18-day observation period, showing how the otherwise cell-repellent POEGMA scaffold could support cell adhesion and proliferation due to the nanofiber structure formed *in situ* around the cells during electrospinning (Xu et al., 2018, 2020).

A few other examples cell electrospinning have been reported using post-crosslinked nanofiber scaffolds, although successful cell electrospinning remains somewhat uncommon. In one such example, C2C12 cells were suspended in a mixture of fibrinogen and the electrospinning aid PEO and electrospun into a collection bath containing thrombin to crosslink the fibers into fibrin (Figure 6B) (Guo et al., 2019). Cell viability, and ultimate differentiation of the myoblasts into mature myotubes, was promoted through the electrospinning process by decreasing the voltage applied to 4.5 kV and by using more stable C2C12 cell aggregates rather than monodispersed cells (Guo et al., 2019). As another example (Figure 6C), C2C12 myoblasts were electrospun in water with alginate and PEO (electrospinning aid) and then crosslinked in a calcium ion/DMEM bath for 2 min to produce hydrogel nanofibers with aligned morphologies (Yeo and Kim, 2018). The C2C12 cells maintained cell viabilities above 90% for 7 days and grew along the directions of the aligned fibers, with a follow-up study showing similar efficacy with HUVEC cells (Yeo and Kim, 2020). Subsequent seeding of C2C12 cells on top of the alginate/PEO/HUVEC fibers resulted in faster myoblast maturation compared to the same scaffold without the HUVECs (Yeo and Kim, 2020). However, the design of electrospinning strategies compatible with cells is still in its infancy despite offering enormous potential to create ECM mimics that can reproduce the nanofibrous internal morphologies of native ECM.

#### 3.2.5 Microfluidic Hydrogel Fibers

While we have focused on the use of electrospinning as a method to enable simultaneous nanofiber production and cell encapsulation, microfluidics can also be used to generate fibers at somewhat larger length scales that, while potentially less directly biomimetic of nanofiber dimensions in native ECM, can still play useful roles in directing cell adhesion and proliferation responses. Hydrogel fibers can be prepared either by spinning hydrogel precursors from a microfluidic head composed of spinneret or capillaries or by using a PDMS microdevice with microchannels (Jun et al., 2014). Compared to electrospinning, the high degree of customization possible in a microfluidic chip can enable the fabrication of multiple types of internal structures inaccessible with other fabrication techniques (Jun et al., 2014; Sun et al., 2018). Cells can be encapsulated by mixing them together with the hydrogel precursors (as with electrospinning) or by merging cell-containing droplets with the fiber precursor droplets directly on-chip, minimizing the time over which the cells and the uncrosslinked precursor materials directly interact and thus potentially reducing any cytotoxicity related to such precursor materials (Guimarães et al., 2021; Wang et al., 2021). As one example of such an approach, Wang et al. fabricated one-step aqueous-droplet-filled hydrogel fibers as islet organoid carriers using a coaxial channel microfluidic chip in which droplets containing human induced pluripotent stem cells (hiPSC) loaded into the inner channel were encapsulated in an alginate hydrogel shell formed from middle and outer channels by ionic crosslinking of sodium alginate (NaA) with calcium chloride (CaCl_2_) (Wang et al., 2021). However, the diameter of fibers fabricated via microfluidic strategies typically ranges from a few micrometers to millimeters, far larger than the size of fibers (hundreds of nanometers to a few micrometers) found in native extracellular matrix (Jun et al., 2014). As such, while microfluidic-produced microfibers offer the potential for more complex morphologies and likely less aggressive chemical/physical fabrication conditions perhaps better suited for processing more sensitive cell lines into fibrous structures, electrospun scaffolds offer significantly better resolution to mimic the nanofibrous structure in native extracellular matrix.

### 3.3 *In Situ* Tissue Engineering

Instead of conventional tissue engineering in which a cellularized scaffold is implanted at the defect site, *in situ* tissue engineering involves the injection of hydrogels (typically but not necessarily including cells) to the defect site to direct regeneration using a minimally-invasive technique (Gaharwar et al., 2020). In this context, the body’s own capacity for regeneration supported by the injected hydrogel that provides the required biophysical and biochemical cues to guide or stimulate functional restoration at the site of damaged tissues and/or deliver viable cells that can be used to create healthy tissue at a diseased tissue site (Pratt et al., 2004; Malek-Khatabi et al., 2020). The use of *in situ-*gelling hydrogels that can crosslink spontaneously upon injection (via physical interactions and/or click/click-like covalent bonding) is most common given that *in situ* gelation facilitates local injection of the hydrogel without the need for transplantation (Dias et al., 2020) (Figure 4). Also, *in situ-*gelling hydrogels can adapt the shape of the hydrogel directly for the defect site in the surgical room, particularly advantageous to treat irregularly shaped injury sites that may be difficult to fill accurately using an *ex vivo*-fabricated scaffold (Dias et al., 2020). The key design criteria for injectable hydrogels useful for *in situ* tissue engineering were described in a recent review by Young et al.: 1) to maintain the viability and function of encapsulated cells; 2) to reproduce the target tissue morphology, including its mechanical profile and adhesion to surrounding tissues; and 3) to tune the degradation rate to ensure that complete tissue replacement can occur on a clinically-relevant timescale (Young et al., 2019). Table 2 summarizes multiple materials and methods used for *in situ* tissue engineering.

**TABLE 2:** Materials and methods used for *in situ* tissue engineering.

Materials	Crosslinking chemistry	*In situ* gelation mechanism	Cells encapsulated	Key biological results	FDA approved constituent materials	References
Alginate and collagen	Calcium sulfate (ionic crosslinking)	Pre-mixed in a 1:1 alginate:collagen volume ratio	Human mesenchymal stem cells (hMSCs)	• >90% viability over 7 days after injection	Yes	Moeinzadeh et al. (2021)
				• DNA content increased up to 37-fold after 28 days		
Chitosan and dextran	Amine-functionalized chitosan crosslinked with aldehyde groups on oxidized dextran (imine covalent bond)	Pre-mixed	Human fetal osteoblasts	• >90% viability after 7 days after encapsulation	Yes	Cheng et al. (2014)
Decellularized ECM and methacrylated hyaluronic acid	Thermal gelation at 37°C followed by *in situ* photocrosslinking	Pre-mixed, intrapericardial injection (iPC)	MSCs, induced pluripotent cardiac progenitor cells (iPS-CPS)	• Cardiac patch increases the cardiac retention of therapeutics and improves cardiac function post-myocardial infarction	Yes	Zhu et al. (2021)
Gelatin-based microribbons (using wet-spinning in DMSO) and fibrinogen	Thrombin-induced crosslinking (enzymatic)	Pre-mixed	Adipose-derived stromal cells (for bone regeneration)	• >80% viability using 16G needle at 5% microribbon density	Yes	Tang et al. (2020)
				• Osteogenic capability post-injection is preserved using staining techniques		
				• Complete degradation after 3 weeks		
Poly (oligoethylene glycol methacrylate)	Hydrazide and aldehyde-functionalized oligomers (hydrazone covalent bond)	Real time mixing from a double barrel syringe through a static mixer	Murine C2C12 myoblast cells	• Cationic-functionalized POEGMA copolymers can deliver viable and proliferating ARPE-19 human retinal epithelial cells	No	Bakaic et al. (2018)
Fibrinogen	Thrombin-induced crosslinking	Pre-mix	hMSCs	• Two-fold increase in cardiac retention coupled with two-fold reduction in liver accumulation	Yes	Martens et al. (2009)

**FIGURE 7:**
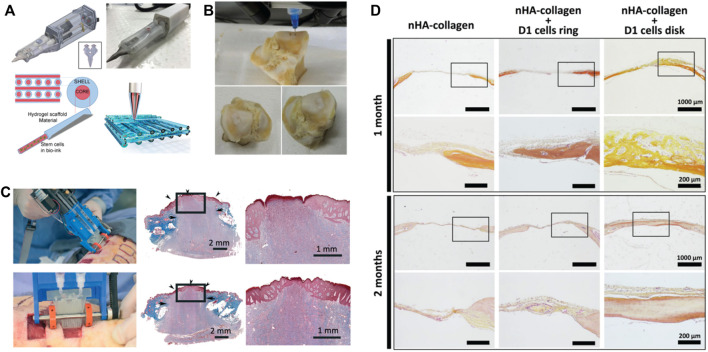
**(A)** Design of the handheld Biopen to print gelatin methacrylamide and hyaluronic acid methacrylate (HA-GelMa) hydrogel scaffolds with core-shell structure. Reproduced with permission (di Bella et al., 2018). **(B)** *In situ* bioprinting to fabricate HA hydrogels for chondral defect repair. Reproduced with permission (Li et al., 2017). **(C)** *In situ* formation of fibrin-HA/collagen sheet for skin tissue regeneration. Reproduced with permission (Hakimi et al., 2018). **(D)** *In situ* bioprinting of mesenchymal stromal cells and nano-hydroxyapatite collagen for *in vivo* bone tissue regeneration. Reproduced with permission (Keriquel et al., 2017).

While the direct injection of the *in situ*-gelling precursor polymers can be performed into any site, there is growing interest in leveraging *in situ* gelation chemistry with 3D bioprinting to perform *in situ* (or *in vivo*) printing (Singh et al., 2020), typically using either a robotic arm or a handheld device that could be used directly by healthcare professionals on the patient and thus eliminates the need for moving the printed tissue between the printer and the operating room (Duchi et al., 2017; di Bella et al., 2018; Hakimi et al., 2018) (Figure 7A–C). Such an approach leverages the reproduction of fine structures enabled by 3D bioprinting while eliminating the need for subsequent transplantation, which is challenging in the context of conventional 3D bioprinting due to 1) the potential for the disruption of both micro and macro-architectures (i.e., from swelling), 2) the challenges around maintaining structural fidelity and integrity upon handling and transport, 3) the risk of contamination and thus the need to maintain a highly sterile environment throughout the entire fabrication/transplant process, and 4) the requirement for good replicability and low error rates. *In situ* bioprinting has already been demonstrated to enable functional tissue regeneration in multiple tissue types including bone, skin, and cartilage. In the following sections, we will highlight key examples of strategies to address these challenges specific to *in situ* 3D bioprinting; however, any of these materials could also be directly injected to enable *in situ* tissue engineering using a more direct injection-based approach.

#### 3.3.1 Bone

Bone defects are conventionally treated using non-biological implants that either fill the missing bone part or support the remaining bone to provide enough mechanical strength for a limited time (Liu et al., 2017). However, such implants do not provide a long-lasting solution and will need to be surgically replaced over time, leading to potential complications. Keriquel et al. (2017) demonstrated the use of an *in situ* laser-assisted bioprinting process as a viable technique for printing mesenchymal stromal cells loaded in a collagen-nano hydroxyapatite hydrogel to promote functional bone regeneration in a murine calvaria defect model. Laser-assisted bioprinting (LAB) uses a near-infrared pulsed laser beam coupled to a scanning mirror and focusing system to enable high cell printing resolution and precision (Keriquel et al., 2017). While the proliferation of the printed cells was not significantly different between the ring and disk geometries tested, the disk geometry showed a significant increase in bone formation after 1 and 2 months (Keriquel et al., 2017) (Figure 7D). Alternately, Cohen et al. (2010) demonstrated the feasibility of *in situ* bioprinting using an extrusion-based bioprinter to print alginate-based hydrogels to treat chondral and osteochondral effected in a calf femur. Alginate was mixed with demineralized bone ECM and gelatin and printed directly into the osteochondral defect, enabling the functional repair of defects directly at the injury site with precise geometry constraints (mean surface errors of <0.1 mm) (Cohen et al., 2010). The inherent mechanical mismatch between bone ECM and a printable hydrogel should however be considered in this context, providing further impetus toward accelerating existing efforts to fabricate hydrogel-based bioinks with a higher modulus value without compromising printability.

#### 3.3.2 Skin

Skin is the largest and most accessible organ and has thus become an ideal platform to validate *in situ* tissue engineering approaches to create skin substitutes, though only a few studies have been reported. Hakimi et al. developed a portable handheld extrusion-based skin printer that allowed for the co-printing of both ionically crosslinked biomaterials (i.e., alginate) and enzymatically crosslinked protein (i.e., fibrin) with dermal and epidermal cells to form cell-laden sheets of consistent thickness, width, and composition (Hakimi et al., 2018) (Figure 7C). The *in situ* deposition was validated in a murine wound model and a porcine full-thickness wound model, with the bioprinted structure observed to cover the full wound with a homogeneous layer that did not impede on normal re-epithelization or wound contraction (Keriquel et al., 2017). Alternately, Albanna et al. (2019) reported a mobile inkjet printer that facilitates the precise delivery of either autologous or allogeneic dermal fibroblasts and epidermal keratinocytes directly into an injured area. Using a thrombin-crosslinked fibrinogen hydrogel and an integrated imaging technology to pre-determine the topography of the wound, the direct bioprinting approach rapidly closed the wound, reduced wound contraction, and accelerated re-epithelialization in a murine full-thickness excisional wound model; the wound area at 1-wk post-surgery was 66% of the original wound area for the printed group compared to 95% of the control group, while the printed skin cells completely closed the wound by 3 weeks post-surgery compared to 5 weeks for the controls (Albanna et al., 2019). Developing strategies that can *in situ* print striated features with thinner dimensions (on the length scale of native skin) may further improve the efficacy of this therapeutic option.

#### 3.3.3 Cartilage

Damaged cartilage cannot be easily treated due to its lack of vasculature and inability to self-repair by our body, with osteoarthritis being the most common chronic joint disease that leads to inflammation and degradation of cartilage (Liu et al., 2017). While existing treatments are available in the form of implantation and grafts, such treatments are highly invasive, expensive, and require revisions over time. Furthermore, the implantation of prefabricated tissue scaffolds often results in a significant geometric mismatch to the native tissue while lacking sufficient structural support and nutrient diffusion. O’Connell created a handheld pneumatic *in situ* extrusion device dubbed the “BioPen” that addresses these challenges by enabling printing of two inks in a collinear geometry (di Bella et al., 2018). High viability (>97%) of human adipose stem cells could be maintained after 1 week using methacrylated gelatin and hyaluronic acid hydrogel bioinks (O’Connell et al., 2016). The design of the BioPen has since been improved by the same group to allow for a multi-inlet extruder nozzle and a motorized extrusion system, permitting coaxial *in situ* bioprinting in which the core bioink encapsulated the stem cells and the shell bioink (mixed with the photoinitiator) (Figure 7A) is cured *in situ* via ultraviolet (UV) photocrosslinking in the chondral defect of a sheep knee joint (O’Connell et al., 2016; Duchi et al., 2017). The *in vivo* 3D-printed construct showed early formation of hyaline-like cartilage and better macroscopic/microscopic characteristics than the controls, with the coaxial mixing approach better protecting the cells from the printing process and any potential damaging effects of the free radicals generated during photocrosslinking (Duchi et al., 2017).

## 4 Clinical Translation of Hydrogels

To be appropriate for clinical translation, a hydrogel must be biocompatible and have suitable mechanics and an appropriate degradation profile while addressing key biological challenges (e.g., effective regeneration while avoiding unwanted immune responses) and logistical challenges (e.g., sterilizability, transportation, and storage) associated with the targeted application. The majority of clinically approved injectable or implantable hydrogels are targeted for skin or joint regeneration or drug delivery applications (Mandal et al., 2020). Table 3 summarizes the various classes hydrogels that have been approved for clinical use. As shown, injectable or implantable hydrogels for cell therapies have not yet been approved for clinical use by the United States Food and Drug Administration (FDA) (Sivaraj et al., 2021); however, one cell-laden hydrogel scaffold has been approved for wound healing applications. Apligraf^®^ is a bovine collagen-I hydrogel scaffold loaded with human neonatal fibroblasts that is covered on one side by a stratified layer of neonatal keratinocytes (Gentzkow et al., 1996). This tissue-engineered graft is approved for the treatment of chronic wounds such as diabetic foot ulcers (DFU) and venous leg ulcers (VLU) (Streit and Braathen, 2000; Edmonds, 2009). The hydrogel graft is typically applied to the wound site for a period of weeks and can be reapplied as needed at the discretion of the physician (Zaulyanov and Kirsner, 2007). A study investigating the persistence of Apligraf^®^ in acute wounds concluded that the DNA from Apligraf’s cells was very minimally detected after 4 weeks and that the hydrogel did not exhibit features of engraftment (Griffiths et al., 2004); however, Apligraf^®^ still shows clinical efficacy, making the cellular secretions of pro-healing cytokines the most likely mechanism of action (Streit and Braathen, 2000; Zaulyanov and Kirsner, 2007). A clinical study with over 100 participants that investigated anti-bovine collagen-I and anti-bovine serum antibodies as well as T-cell proliferation found no humoral or cellular immune response to Apligraf^®^ in comparison with the control group (Eaglstein et al., 1999), suggesting its safety. Decellularized ECM scaffolds combined with cells have also been clinically approved for soft tissue repair (e.g., AlloDerm^®^, GRAFTJACKET Now^®^, and OrthADAPT^®^ Bioimplant) but are generally very expensive given the extensive purification steps required to ensure sufficiently consistent batch-to-batch manufacturing of non-inflammatory decellularized matrix. A cell-loaded alginate-based matrix is the only currently non-ECM derived approved tissue regeneration hydrogel (AlgiMatrix^®^) but to-date is only used for 3D tissue models for *in vitro* drug screening. As such, despite the massive developmental efforts invested to-date in the design and fabrication of tissue engineering-based hydrogels, the translation of such hydrogels remains slow.

**TABLE 3:** Commercialized hydrogels used in the clinic.

Materials	Product name	Crosslinking mechanism	Cell/tissue types	Application(s)	Clinical use	Comments	References
Collagen	Apligraf ^®^	Physical	Fibroblast and keratinocytes	Tissue regeneration, wound healing	Diabetic foot ulcers and venous leg ulcers (VLUs)	Immunologically inert	(Gentzkow et al., 1996b; Helary et al. (2012)
	AlloDerm^®^	Decellularization	Fibroblasts, epithelium, and blood vessels	Soft tissue regeneration	Breast reconstruction	Free of inflammatory response, expensive	Agarwal et al. (2015); Patel et al. (2021)
	GRAFTJACKET Now^®^	Decellularization	Multiple cells	Tissue regeneration	Tendon and ligamentous tissue	Free of inflammatory response	Snyder et al. (2009)
	OrthADAPT^®^ Bioimplant	Decellularization	Multiple cells	Soft tissue regeneration	Attachment of tissue to bone, tendon repair	A highly organized Type I collagen scaffold provides high mechanical strength	Weil (2008); Rana et al. (2017)
	Permacol^®^	Decellularization and chemical crosslinking (hexamethylene diisocyanate)	N/A	Soft tissue regeneration	Tendon and ligament repair, surgical implant for ventral hernia repair and abdominal wall reconstruction	Long-lasting dimensional stability ensures the integrity of the scaffolds	Linz et al. (2013)
	TissueMend^®^	Decellularization	N/A	Soft tissue replacement	Tendon and ligament repair	Potential immune response and lack of long-term mechanical stability	Chen et al. (2009)
	Collagen Implant, CosmoDerm^®^	Decellularization	N/A	Soft tissue augmentation	Skin tissue engineering	Lack of biological function and mechanical stability	Bauman (2004)
	Zimmer^®^ Collagen Repair Patch	Decellularization	N/A	Soft tissue regeneration	Rotator cuff and tendon repair	Good cell penetration and vascularization, lack of long-term stability	Coons and Alan Barber (2006)
Alginate	AlgiMatrix^®^	Physical	Multiple cells	3D cell culture	3D cell/tissue culture models (e.g., tumor models)	Good cell morphology and differentiation supported	Godugu et al. (2013)
	Gel-One^®^	Chemical	N/A	Injectable soft tissue fillers	Treatment of osteoarthritis, reducing pain scores	Lack of stiffness and long-term mechanical stability	Ishikawa et al. (2014)
Hyaluronic acid (HA)	Hyaloglide^®^	Chemical	N/A	Injectable tissue spacers and adhesive	Prevent or reduce adhesions or fibrosis after tendon, peripheral nerve, or articular surgery	High viscosity, lower cost than ECM/dECM options	Riccio et al. (2010)
	Juvéderm^®^	Chemical	N/A	Soft tissue augmentation	Dermal wrinkles and folds	Side effects and expensive	Allemann and Baumann (2008); Barr et al. (2015)
	Hyalonect^®^	Chemical	N/A	Tissue regeneration	Orthopedic and trauma reconstructive surgeries	Low inflammatory response	Rhodes et al. (2011); Tekin (2013)
	Veriset^®^ hemostatic patch	Chemical	N/A	Hemostat	Intraoperative soft tissue bleeding	Consistent efficacy across multiple tissues	Glineur et al. (2018)
Poly (ethyl-ene glycol) (PEG)	DuraSeal^®^	Chemical	N/A	Sealant	Prevent cerebrospinal fluid (CSF) leakage after cranial and spinal surgery	Potential long-term issue with postoperative cord compression	Lee et al. (2013)
	SpaceOAR^®^ hydrogel	Chemical	N/A	Soft tissue spacer	Prostate cancer rectal spacer to reduce the radiation dose delivered to the anterior rectum	Transparent and expensive with low side effects	Hedrick et al. (2017)
Polyacryl-amide (PAM)	Bulkamid^®^	Physical	N/A	Injectable gel fillers	Stress urinary incontinence treatment	Long-term stability, some potential tissue side effects	Kasi et al. (2016)

Clinically approved hydrogel therapies that do not include cells fall into four broad categories: natural hydrogels for cosmetic applications (Busso and Voigts, 2008; Lorenc, 2012; Sparavigna et al., 2014), natural or synthetic hydrogels for functional volume-filling purposes (Sack et al., 2018; Freitas et al., 2020), natural or synthetic hydrogels for osteoarthritic pain management (Miltner et al., 2002; Tnibar et al., 2015), or synthetic hydrogels for sustained drug delivery (Shore et al., 2011). The characteristics of such clinically approved hydrogels can serve as relevant case studies for understanding the regulatory and translational considerations that may be limiting for the translation of tissue engineering hydrogels. We will discuss two such examples: Juvéderm^®^ and Bulkamid^®^.

Juvéderm^®^ is a 1,4-butanediol diglycidyl ether (BDDE)-crosslinked hyaluronic acid (HA) gel clinically approved in 2006 in the United States for aesthetic treatment of dermal wrinkles and folds (Allemann and Baumann, 2008; Barr et al., 2015). Pre-clinical studies of chemically crosslinked HA showed that its degradation profile (Sall and Férard, 2007; Boulle et al., 2013) and the cytocompatibility of its degradation by-products (RD, 1996; Boulle et al., 2013) are suitable for clinical applications; following, numerous clinical trials have concluded that HA dermal fillers have significant advantages in longevity and biocompatibility over other treatment options (Duranti et al., 1998; Sparavigna et al., 2014; Barr et al., 2015). Slight variations of Juvéderm with different degrees of crosslinking and therefore different stiffnesses and longevity have also been approved and widely adopted for clinical use (Allemann and Baumann, 2008). Clinical translation of Juvéderm was bolstered by the fact that hyaluronic acid had already been used clinically for decades as a vitreous substitute (Pruett et al., 1979) as well as the approach to manufacture HA via bacterial fermentation processes, improving the batch-to-batch consistency of the HA product.

Bulkamid^®^ is an injectable synthetic polyacrylamide hydrogel approved by the FDA for use in the United States in 2020 to treat stress urinary incontinence (SUI) by functioning as a bulking agent to add a total volume of up to 2 ml to the walls of the urethra (U.S. Food and Drug Administration, 2021; Kasi et al., 2016). *In vitro* and *in vivo* studies investigated the cytotoxicity (McCollister et al., 1965) and degradation (Caulfield et al., 2002) of polyacrylamide, informing the choice of the 2.5% (w/v) polyacrylamide concentration that was confirmed via multiple long-term clinical studies to be suitable in the targeted application (Lose et al., 2010; Pai and Al-Singary, 2015; Kasi et al., 2016). Although polyacrylamide was not previously approved for clinical use by the FDA, it had been in clinically used for breast augmentation in China since 1997 and years earlier in Ukraine (Margolis et al., 2015), albeit with reports of multiple adverse complications such as lumps, breast pain and induration, and in certain cases a foreign body response which destroyed the structure of adjacent muscle and gland (Qiao et al., 2005). The much smaller volume of gel required for Bulkamid injection (total of 2 ml in 3 to 4 locations) relative to breast injections (∼150 ml) and the structure of the urethra significantly suppresses this risk, even in the context of a non-degradable hydrogel like polyacrylamide.

In both cases, the clinical translation of Juvéderm and Bulkamid benefited from the pre-existing clinical use of the same hydrogel for a different application, motivating translational researchers to focus efforts on developing variations of hydrogels that are already approved for clinical use; this tendency is strongly reinforced by the high costs of funding a therapy from pre-clinical studies through to FDA approval. These barriers hinder innovative hydrogel formulations from entering the process of clinical translation, particularly in the area of tissue engineering in which both a material and a therapeutic (i.e., a regenerative cell) are key parts of the functional product. These barriers, and the regulatory challenges in dealing with regenerative tissue scaffolds, have been recognized by the FDA, which in 2017 acknowledged that as we enter a “new era of 3D printing of medical products regulatory issues related to the bioprinting of biological, cellular and tissue-based products” will have to adapt as the applications and practical implementations of tissue engineering continue to drift from the definitions of traditional therapies (U.S. Food and Drug Administration, 2017). This emerging regulatory framework must be navigated by researchers in this area with an emphasis on ensuring the manufacturability, sterilizability, and storage stability of any hydrogel formulation developed in addition to ensuring efficacy of the product for the intended application.

While the majority of discussion on translation has focused on the materials design, the cell sourcing and production of large number of functional cells for practical translation is perhaps an even more challenging problem given the limited potential of many cell types to be expanded in a lab and the potential for non-host transplants to induce potential immune response for patients. The ability of tissue engineering scaffolds to fully recapitulate the full complexity of the microenvironment some more sensitive cells need to function is also to-date weakly addressed in the area, at least using scalable methodologies for scaffold manufacturing. While not the main focus of this review, it is critical that methods to use the patient’s own cells (e.g., induced pluripotent stem cells from patients that can be reprogrammed to the desired phenotype) and/or to expand immune-compatible cell lines are developed and scaled to ensure consistency in the cell responses and thus reliable tissue regeneration responses.

## 5 Conclusion and Outlook

Hydrogels as tissue engineering scaffolds offer enormous potential for clinical translation given their clear advantages in maintaining a hydrated environment for cells, mimicking the mechanics of soft tissues, and facilitating the presentation of appropriate interfaces and cell cues to promote tissue regeneration. However, the slow translation of these materials to the clinic goes beyond simple regulatory barriers. For natural polymer-based hydrogels, the effective scale up and management of batch-to-batch differences in natural polymers remains a huge challenge, as is addressing the often weaker mechanical properties of natural polymer-based hydrogels for promoting proper signaling to regrow stiffer tissues. For synthetic polymer-based hydrogels, challenges around degradability can be limiting (although the approval of Bulkamid suggests that this challenge is not inherently problematic for regulatory approval but rather related to the need to balance scaffold clearance with the rate of tissue regeneration). Combining natural and synthetic polymers into a single scaffold may offer benefits to leverage the benefits (and dilute the drawbacks) of each individual polymer type, although the inclusion of multiple materials is also likely to further complicate the regulatory process.

The three techniques described in depth that enable scaffold fabrication and cell loading in a single step (3D bioprinting*,* cell electrospinning, and *in situ* tissue engineering) in our view offer the most practical ways forward to commercialize cell-loaded tissue engineering hydrogels given that they all streamline multiple fabrication steps, make maintaining sterility much easier, and could in principle be applied directly in the operating room. However, each method still offers challenges, particularly in terms of broadening the types of materials that can be used for scaffold fabrication. For bioprinting, a relatively small number of bioink options is currently available due to the requirements of printing and the need to maintain precise crosslinking kinetics to fix the printed shape in place, although the emergence of embedded printing approaches (e.g., FRESH) can help to offset some of these challenges. The still relatively low feature resolution printable with high-throughput printing strategies like extrusion also offers a limitation of current bioprinting strategies for reproducing the nanofibrous structure of native ECM. Cell electrospinning can provide similar feature sizes to native ECM (i.e., in a range of hundreds of nanometers to a few micrometers) essential to provide cues for cell adhesion and proliferation but involves the exposure of cells to a strong electric field and (in typical processes) induces at least some degree of cell dehydration as the electrospun scaffolds are collected, both of which apply mechanical and/or osmotic stresses on cells that may compromise the viability of particularly more sensitive primary cell lines. Electrospinning directly on tissues (e.g., using hand-held devices) or into cell media instead of on dry collectors may address some of these challenges, while minimization of the applied voltage can ensure as high as possible cell membrane viability during the electrospinning process. *In situ* tissue engineering approaches offer a mechanism to directly administer cells and hydrogels into the body via injection but are more difficult to fabricate into controlled internal microstructures and morphologies optimized for cell growth. Methods to perform *in situ* gas foaming using biologically-safe foaming agents, incorporate sacrificial pore-forming materials that can be dissolved or otherwise *in situ* degraded over time to introduce tunable porosity, and/or provide alignment cues (e.g., via the incorporation of anisotropic nanoparticles that can be remotely aligned via non-invasive stimuli (de France et al., 2017)) all offer potential to improve the outcomes of such therapies, although further research is certainly required to make such approaches truly translatable. While each of these three processes offers in our opinion outstanding promise for translation, further developments in these processes (or the development of new processes) that place minimal stress on cells and can be used with the broadest possible set of materials would further expand the potential for practical translation of hydrogel-based tissue scaffolds to the clinic.
